# EpCAM aptamer modified carbon@manganese dioxide nanoflower for synergistic drug-nanozyme allergic rhinitis treatment

**DOI:** 10.1016/j.mtbio.2026.103588

**Published:** 2026-08-26

**Authors:** Leichao Zhang, Longying Cong, Yiyao Gao, Wei Zhang, Yiting Liu, Lingyu Zhang, Qian Xiu

**Affiliations:** a Department of Pathology, China-Japan Union Hospital of Jilin University, 126 Xian Tai Street, Changchun, Jilin, 130000, PR China; b Department of Scientific Research Center, China-Japan Union Hospital, Jilin University, 126 Xian Tai Street, Changchun, Jilin, 130000, PR China; c Department of Chemistry, Northeast Normal University, 5268 Ren Min Street, Changchun, Jilin, 130024, PR China; d Department of Otolaryngology-Head and Neck Surgery, China-Japan Union Hospital, Jilin University, 126 Xian Tai Street, Changchun, Jilin, 130000, PR China

**Keywords:** Allergic rhinitis, Nanozyme, Targeted drug delivery, Budesonide, Reactive oxygen species (ROS), EpCAM aptamer

## Abstract

Allergic rhinitis (AR) is a prevalent upper respiratory allergic disorder that significantly impacts patients' quality of life.In this research, we developed a multifunctional nanozyme-drug synergistic therapy nanosystem for the treatment of allergic rhinitis (AR). The nanosystem, called EpCAM aptamer modified carbon@manganese dioxide nanoflower (Apt-C@MnO_2_ NFs), combines porous carbon nanospheres and MnO_2_ nanozymes to achieve synergistic reactive oxygen species (ROS) scavenging. EpCAM aptamer (Apt) is an epithelial cell adhesion molecule. The designed material was modified with EpCAM aptamers, endowing it with prominent targeting capability to epithelial cells. The modification with PEG facilitates the penetration of NFs through mucus more rapidly, and the Apt enables the NFs to specifically target human nasal epithelial cells (HNEPc). In addition, it can effectively load and sustainably release the anti-inflammatory drug budesonide (BUD). *In vitro* experiment demonstrated the system's ability to target HNEPc, deliver drugs, exhibit multi-enzyme activity, and regulate inflammatory factors. *In vivo* studies in mice showed that Apt-C@MnO_2_ NFs could restore the expression of mitochondrial biogenesis-related proteins, reduce inflammatory cell infiltration, regulate inflammatory pathways, and alleviate AR symptoms. The research provides a new approach for AR therapy, offering a multifunctional system with significant anti-inflammatory effects and imaging tracking capabilities. This innovative therapeutic strategy combines nanozyme technology with targeted drug delivery to achieve effective treatment of AR.

## Graphical abstract

Apt-C@MnO_2_ NFs have the characteristics of targeting epithelial cells, drug delivery, multi enzyme activity, and regulating inflammatory pathway, and have an inhibitory effect on inflammation, finally alleviate AR symptoms.

**Figure:**
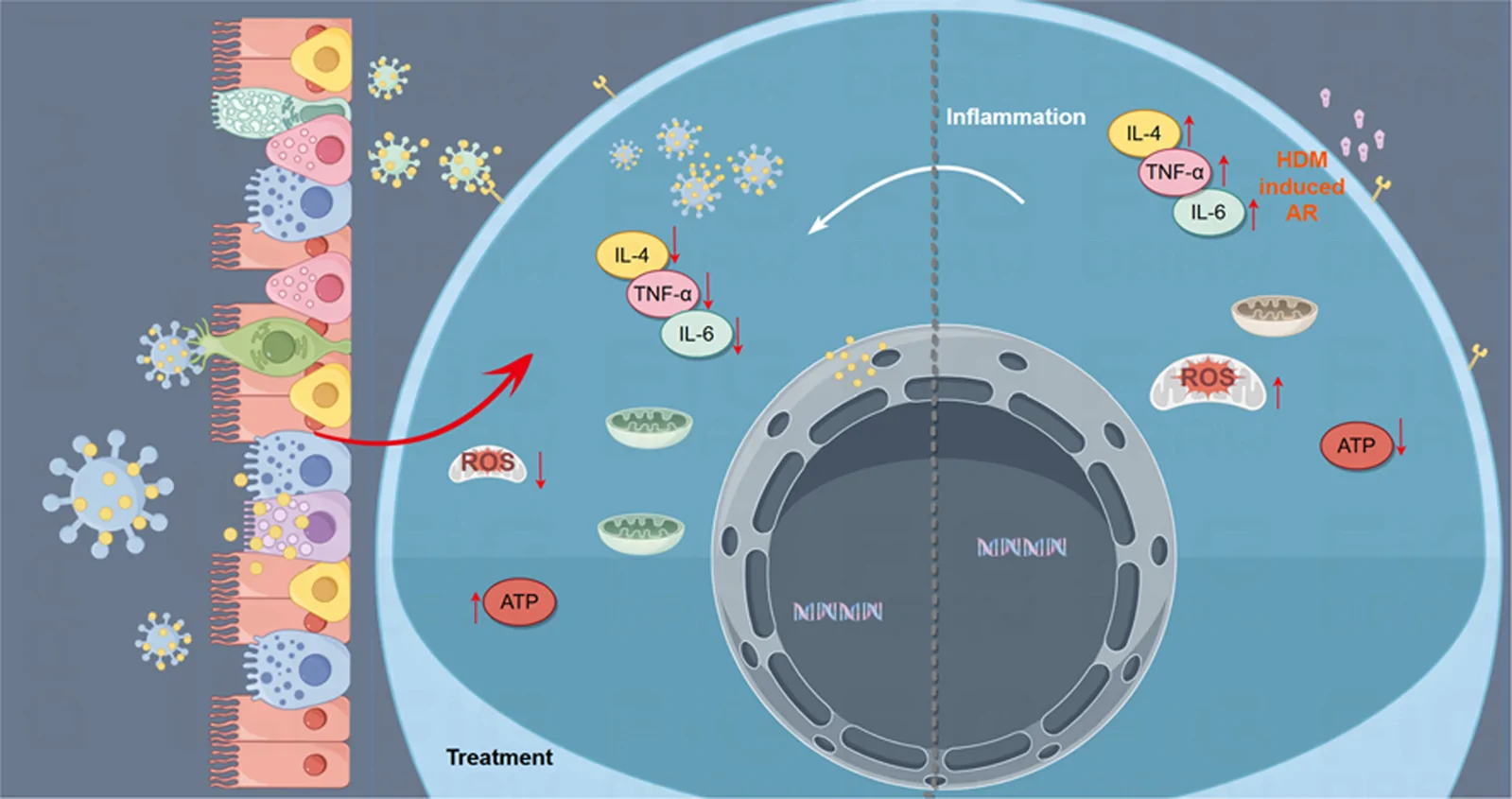

## 1 Introduction

Allergic rhinitis (AR) is a common upper respiratory allergic disease which activated when immunoglobulin E (IgE) and helper T cells (Th2) detect allergens inhaled from the environment [1]. The immune response of AR can be provoked by various allergens: dust mites are one of the most common allergens worldwide, affecting approximately 50 % of AR patients [2,3]. AR may cause nasal irritation, congestion, and sneezing [4]. As previously reported, the reactive oxygen species (ROS) level in the nasal mucosa of AR mice was higher than that in the normal mice [5], accompanied with increasing inflammatory cells [6] (such as eosinophils and goblet cells) and upregulating pro-inflammatory cytokine levels [[7], [8], [9], [10], [11]]. High levels of ROS can disrupt the redox balance within an organism, exceeding the capacity of the cellular antioxidant defense system, leading to oxidative stress [12] and irreversible tissue damage. Mitochondrial biogenesis occurs in certain small vascular smooth muscle cells in asthma and in mononuclear inflammatory cells in the alveolar region [13], which is a normal response after cell damage [14]. As the master switch governing mitochondrial biogenesis, PGC-1α [15] activates nuclear and mitochondrial gene expression and modulates fission proteins to control mitochondrial fission; upon energy deprivation, AMPK triggers PGC-1α-driven mitochondrial biogenesis to boost cellular energy output, while nuclear-encoded TFAM [16] translocates into mitochondria to bind mtDNA, initiate its replication and transcription, and preserve mtDNA stability. Other respairytory diseases such as: lung injury [17] lead to uncontrolled inflammatory response and excessive generation of ROS.Additionally, inflammation may disrupt mitochondrial biogenesis, leading to impaired mitochondrial function. Therefore, effective clearance of ROS and restoration of mitochondrial function can alter the internal microenvironment of the nasal cavity and alleviate AR and it may exert therapeutic effects on oxidative stress induced by the treatment of other respiratory diseases. Generally, AR can be treated with both steroids and non-steroidal anti-inflammatory drugs.

However, these anti-inflammatory drugs may cause side effects such as epistaxis, burning, mild irritation, dry mouth, drowsiness, occasional nosebleeds, and dry nose. In addition, due to the short contact time between the drug and nasal mucosa, as well as the influence of nasal cilia and mucus, the drug cannot be fully absorbed before clearance [[9], [10], [11]].

To overcome these obstacles, the development of nanotechnology has provided new hope for the treatment of AR. Previous studies have revealed that certain nanomaterials [18] [19] possess reactive oxygen species (ROS) scavenging and anti-inflammatory functions. The small size and special surface characteristics of nanozymes enable them to penetrate cell membranes, act on inflammatory sites, and effectively clear ROS, thereby reducing the release of inflammatory mediators. Moreover, precise drug delivery by loading anti-inflammatory drugs into specific nanozymes with a target agent is an innovative therapeutic strategy for effective treatment of AR. An active targeting strategy is promising for the delivery of nanocarriers to specific sites. Among these, small strands of DNA or RNA called aptamers have been used as suitable targeting agents because of their high binding affinity, stability, low cost of no immunogenicity [20]. The epithelial cell adhesion molecule, EpCAM aptamer (Apt), is a protein highly expressed on the surface of epithelial cells, with a molecular weight of 39-42 kDa [21,22], some nanomaterials incorporate EpCAM aptamers as a component of chimeras to target tumor cells and boost the cytotoxicity against tumor cells [23,24]. Thus, the Apt can be considered a good target for modifying the surface of a nanozyme for targeted AR treatment. Apt plays an important regulatory role in the anti-inflammatory effects of epithelial cells and can participate in the adhesion between epithelial cells. It can promote tight junctions between epithelial cells and maintain the integrity and stability of the epithelial layer by binding to the Apt on the surface of surrounding cells [25].

Carbon nanozymes, which rely on their inherent enzyme-like activity and unique physicochemical properties, have rapidly emerged as multifunctional nanoplatform. Compared with natural enzymes, carbon nanozymes are more stable, economical, and have adjustable catalytic performance [26,27]. Moreover, carbon can also be used as a supporting material for to integration with other nanozymes for more effective catalysis. Manganese (Mn) is an essential micronutrient in the human body and plays an important role in maintaining the immune system, regulating blood sugar balance, promoting bone growth, reducing inflammation, maintaining metabolic processes, clearing free radicals, maintaining hormone levels, and promoting wound healing [28,29].Manganese dioxide (MnO_2_) has advantages such as controllable size, large surface area to volume ratio, and repeatability in synthesis. It can not only be used as an effective diagnostic imaging contrast agent but can also be modified to deliver drugs to the target site. Thus, the combination of the two nanozymes can achieve a more efficient anti-inflammatory treatment.

**Scheme 1:**
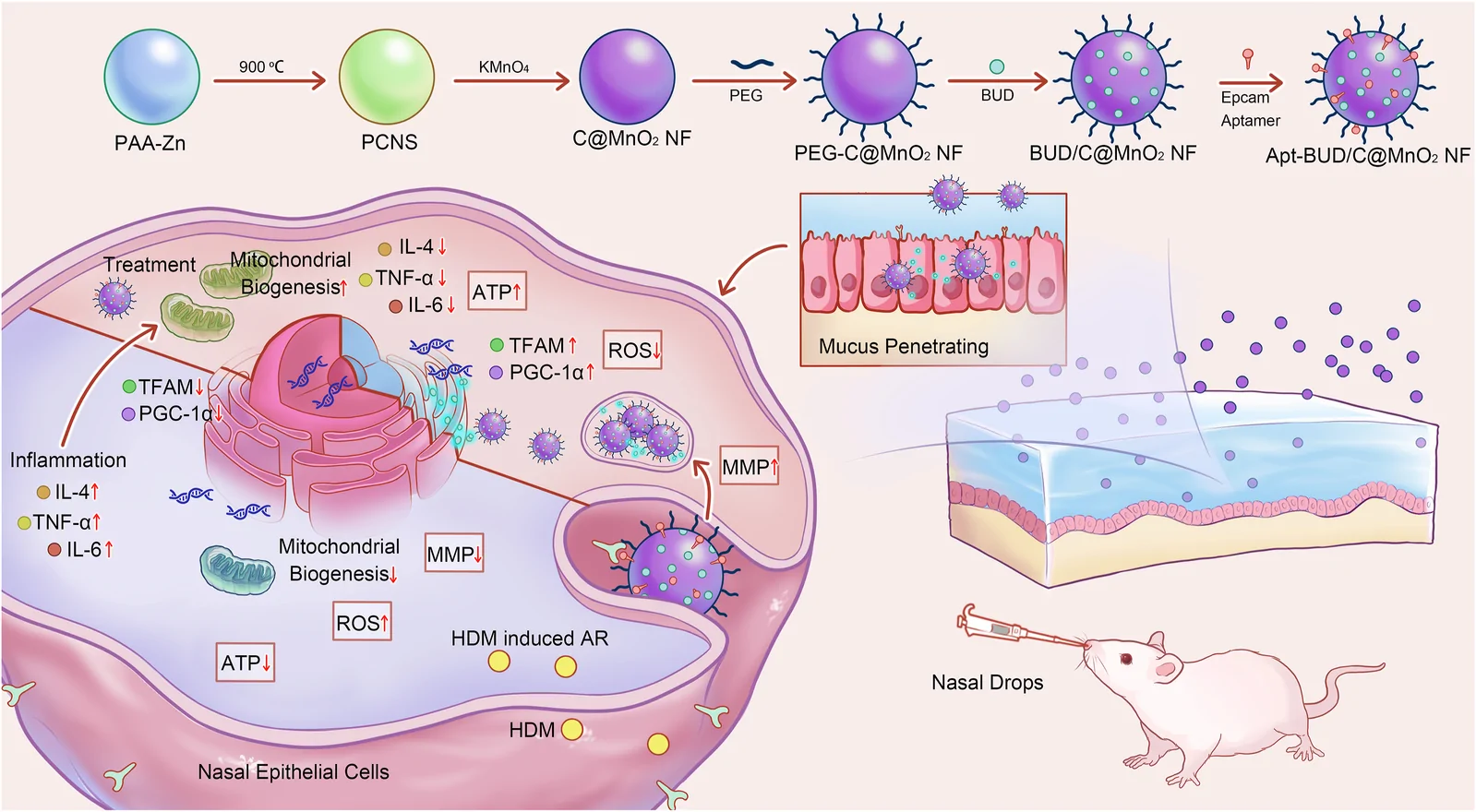
Scheme illustration of the synthesis and application on AR treatment by Apt-BUD/C@MnO_2_ NFs.

Herein, we developed a simple and replicable strategy for constructing a multifunctional nanozyme-drug synergistic therapy nanosystem, Apt modified carbon@manganese dioxide nanoflower (Apt-C@MnO_2_ NFs), which can target human nasal epithelial cell (HNEPc) and is used to treat AR (Scheme 1). As part of C@MnO_2_ NFs, both PCNSs and MnO_2_ NFs have the ability to clear ROS, and this combination improves the multi-enzyme activity of the C@MnO_2_ NFs. Modifying Poly (ethylene glycol) (PEG) on C@MnO_2_ NFs accelerates the smooth penetration of nasal mucus, whereasApt modification targets HNEPc. Benefiting from the pores in carbon, Apt-C@MnO_2_ NFs can load and continuously release the anti-inflammatory drug budesonide (BUD) for synergistic AR therapy. Finally, Apt-BUD/C@MnO_2_ NFs were successfully passed through the nasal mucus and targeted to HNEPc via intranasal administration. After entering the cells, Apt-BUD/C@MnO_2_ NFs harmonized Th2 and Th17 related complex inflammation and oxidative damage induced by house dust mites. Not only that, but also influence mitochondria function and regulate ATP metabolic process in the HNEPc because of the synergistic ROS eliminating effect and BUD, the symptoms of AR mice were alleviated, proving that the BUD loaded Apt-C@MnO_2_ NFs have great potential for AR treatment.

## 2 Materials and methods

### 2.1 Synthesis of PCNSs

1.8 mL polyacrylic (PAA) aqueous solution (0.2 g/mL) was added into 300 mL deionized water, stirred for 15 min, then added 72 mg zinc oxide (ZnO), shook and continued ultrasonic for 10 min until the solution was clarified. 600 mL isopropyl alcohol (IPA) was added to the mixture with a drop funnel and was magnetically stirred overnight to form PAA-Zn NPs [1]. PAA-Zn NPs was centrifugally dried and calcined in a tube furnace at 900 °C for 3 h to obtain PCNSs.

### 2.2 Synthesis of C@MnO_2_ NFs

In an oil bath of 80 ^°^C, 20 mg PCNSs dispersed in 5 mL aqueous solution was added drop by drop into 65 mL KMnO_4_ solution (0.01 mol/L), stirred for 1 h, and C@MnO_2_ NFs were obtained after centrifugation and washing.

### 2.3 Synthesis of Apt-C@MnO_2_ NFs

1 mg C@MnO_2_ NFs and 1 mg NH_2_-PEG-COOH were added into 1 mL aqueous solution, mixed evenly by ultrasound, and mixed on a vertical mixer for 12 h. Then 1 mg EDC and 1 mg NHS was added into the ice bath for 30 min. Then, Apt containing amino groups (5 ′- NH2CACTACAGAGGTTGCGTCTGTCCCACGTTGTCATGGGGGGTTGGCCTG - 3′) [30]as added into above solution, and placed in a vertical mixing apparatus on 4 °C mix for the night. Finally, we centrifuged to obtain Apt-C@MnO_2_ NFs.

### 2.4 Gel electrophoresis

10 μL of Marker, C@MnO_2_ NFs, Apt and Apt-C@MnO_2_ NFs were added to different wells, and then 2 μL 6×electrophoresis sample loading buffer was added to each test hole. The parameters of the electrophoresis instrument are 100 V, 120 mA at constant pressure. After 30 min, the position and strength of the molecules on the gel were recorded by the developer.

### 2.5 Scavenging of ·OH

FeSO_4_•7H_2_O and H_2_O_2_ were added to deionized water, then salicylic acid (SA) was mixed into the above mixed solution and stirred at room temperature for 30 min. Then different concentration of Apt-C@MnO_2_ NFs was mixed into the solution and stirred for 1h. In addition, control groups should be prepared. SA was dissolved in deionized water, and 3 groups of SA solution are taken respectively, and 1 μL H_2_O_2_ was added to one group and FeSO4•7H_2_O was added to the other group. The absorbance of the supernatant at 510 nm was measured by ultraviolet spectrophotometer. Finally, the scavenging ability of Apt-C@MnO_2_ NFs on ·OH was evaluated by comparing the absorbance of the supernatant.

·OH was firstly produced by Fenton reaction, and ·OH was captured by DMPO: 10 μL of H_2_O_2_ (3 %), FeSO_4_•7H_2_O (0.4 mmol/L), DMPO (0.1 mol/L) and sample or distilled water were quickly mixed. The electron paramagnetic resonance (ESR) spectrum of ·OH at 1 min was detected in quartz capillary.

### 2.6 Scavenging of O_2_•-

Riboflavin (20 μM), methionine (13 mM), and NBT (75 μM) were mixed in deionized water (20 mL) to produce a reactant with characteristic absorption at 560 nm. Subsequently, different concentrations of Apt-C@MnO_2_ NFs were added to the reaction solution, and the photoreduction inhibition rate was determined after 15 min of ultraviolet irradiation. Test the absorbance of the supernatant of different samples. The ability of Apt-C@MnO_2_ NFs to remove O_2_^.-^ was evaluated by comparing the absorbance value change.

### 2.7 Scavenging of H_2_O_2_

The absorbance values of the characteristic absorption peaks of H_2_O_2_ in the test samples of different concentrations were determined, and the produced O_2_ was detected at the same time.

### 2.8 Mucus barrier model was established

(1) Configuration of artificial mucus: 500 mg DNA, 250 μL 50 % yolk emulsion, 250 mg mucin, 0.295 mg DTPA, 250 mg NaCl, 110 mg KCl, 1 mL RPMI were added into 50 mL water, ultrasonic until completely dissolved, and preserved at 4 °C.
(2) 5 g gelatin was added into 50 mL water and microwave until melted.
(3) 800 μL liquid gelatin was added into two clean glass bottles, cooled and solidified. Then 1.2 mL mucus was added, and 100 μL (0.5 mg/mL) Apt-C@MnO_2_ NFs and C@MnO_2_ NFs were added by a dropper at top, respectively. Taking photos every 10 min to record the phenomenon. After 40 min, removed the supernatant, heated slightly to melt the gelatin layer, and measured the ultraviolet absorbance of the gelatin layer.

### 2.9 Cell culture

HNEPc were cultured at 37 °C, in a 5 % CO_2_ environment with Dulbecco's modified eagle medium (DMEM) supplemented with 10 % fetal bovine serum (FBS).

### 2.10 *In vitro* cytotoxicity

First, HNEPc was grown on 96-well plates for 24 h. Then, serum-free DMEM containing Apt-C@MnO_2_ NFs (100 - 6.25 μg/mL) was added, respectively. After 24 h, 10 μL MTT was added to each well, continued incubation for 3 h, and then the medium was removed and 150 μL DMSO was added. Absorbance was measured at 560 nm, the survival rate of HNEPc was calculated using equation 1:The percentage of viability is calculated as follows: Cell viability (%) = Absorbance sample/Absorbance control × 100.

### 2.11 Intracellular ROS and mitochondrial detection

In a confocal dish, HNEPc was added and incubated for 24 h. After the culture medium was removed, 200 μg/mL HDM prepared with serum-free culture solution was added except blank control group and incubated for 24 h. After that, Apt-C@MnO_2_ NFs dissolved in serum-free DMEM (25 μg/mL) was replaced and incubated for 12 h and the other groups were replaced with serum-free DMEM. Then DCFH-DA (0.01 mM) in serum-free DMEM and Hoechst 33342 (1 μg/mL) was added to each dish in darkness and incubated for 30 min in sequence. For mitochondrial detection, the dye changed to green fluorescent Mito-tracker, and for mitochondrial membrane potential monitoring, only JC-1 was added. Finally, confocal laser scanning microscope (CLSM) was performed.

### 2.12 Determination of SOD activity, MDA content and protein chip

In the 6-well plate, HNEPc was treated the same as above process before dying. The cells in different groups were lysed and detected according to the instructions of the SOD kit and MDA kit. And the supernatant was collected for testing on a protein chip.

### 2.13 ATP test

HNEPc was planted in 12-well plates for 24 h. 200 μg/mL HDM was added except the control group for another 24 h, then 5 μg/mL BUD solution, 25 μg/mL C@MnO_2_ NFs, 25 μg/mL BUD/C@MnO_2_ NFs, and 25 μg/mL Apt-BUD/C@MnO_2_ NFs were added for 12 h, respectively. The level of ATP was detected by the ATP kit.

### 2.14 Cell targeting experiment

HNEPc and Hela cells were seeded in confocal dishes respectively and incubated for 24 h. Then 25 μg/mL of RITC/C@MnO_2_ NFs was added to HNEPc; RITC/FITC/Apt-C@MnO_2_ NFs was added to HNEPc and Hela cells, respectively. Then the cell nuclei were stained with Hoechst 33342 and imaged by CLSM.

### 2.15 Hemolytic test

Centrifuge the fresh blood, suck out the serum and throw it away. Then the blood is dispersed with NaCl solution, and then centrifuged, repeated operation several times to make the fresh blood supernatant colorless and transparent. Then absorb the solution 200 μL and dilute it with NaCl to 10 mL for use. Finally, Apt-C@MnO_2_ NFs was added to the blood to dilute the final Apt-C@MnO_2_ NFs concentration from 400 μg/mL to 6.25 μg/mL. In addition, the washed blood was dissolved in 1 mL of water as a positive control and dissolved in 1 mL of NaCl as a negative control. The above nine groups of solution were incubated in a water bath at 37 °C for 2 h. Finally, the supernatant was centrifuged for 10 min at a rotating speed of 6000 r/min, the status was recorded by taking photos, and the supernatant was placed in a quartz dish. The absorbance at the wavelength of 540 nm was tested by ultraviolet spectrophotometer, and the hemolysis rate was calculated by the formula according to the measured data.

### 2.16 In vivo experiment

All animal procedures were performed in accordance with the guidelines for Care and Use of Laboratory Animals of Northeast Normal University (202302034). C57BL/6J male mice aged 6-8 weeks with a mass of 18-20 g were selected and kept in an SPF environment for one week, and then randomly divided into 6 groups with 6 animals per group for different treatments: (1) Control, (2) HDM, (3) BUD, (4) C@MnO_2_ NFs, (5) BUD/C@MnO_2_ NFs, (6) Apt-BUD/C@MnO_2_ NFs.

The AR mouse model sensitized by dust mites (HDM) was constructed. In addition to the blank control group with 10 μL pure PBS solution, mice were intranasal with 10 μL PBS solution containing 1 μg HDM protein for 3 days, and then the mice were fed normally for 1 week after the sensitization process. Afterwards, mice were intranasal exposed to 10 μL of PBS in Control group and HDM (10 mg/mL) in other groups, respectively. Subsequently, the therapeutic groups were intranasal instilled with BUD (2 μg/mL), C@MnO_2_ NFs (10 mg/mL), BUD/C@MnO_2_ NFs (10 mg/mL), and Apt-BUD/C@MnO_2_ NFs (10 mg/mL), respectively. The treatment was repeated every day for 5 days. At day 15, blood was collected for protein chip and blood examination, and nasal mucous membrane for SEM, hematoxylin-eosin (H&E) staining, Periodic Acid-Schiff (PAS) stain and Immunohistochemical staining.

### 2.17 Histological analysis and immunohistochemistry

Paraffin sections were stained with HE and then observed by two independent physicians. All monoclonal antibodies (anti-PGC1α, anti-mtTFA) were purchased from Novus(CO,USA). Primary antibodies were applied for 24 h at 4 ^°^C, and slides were rinsed 3 times with phosphate-buffered saline (PBS) prior to incubation with the appropriate secondary antibodies for 2 h. After further washing, the slides were stained with diaminobenzidine (DAB; Maixin, Fujian, Fuzhou, China) and counterstained with hematoxylin. Image Pro-Plus 6.0 software was used to analyze images.

## 3 Results and discussion

### 3.1 Complex changes of inflammation and mitochondria function in AR

**Fig. 1:**
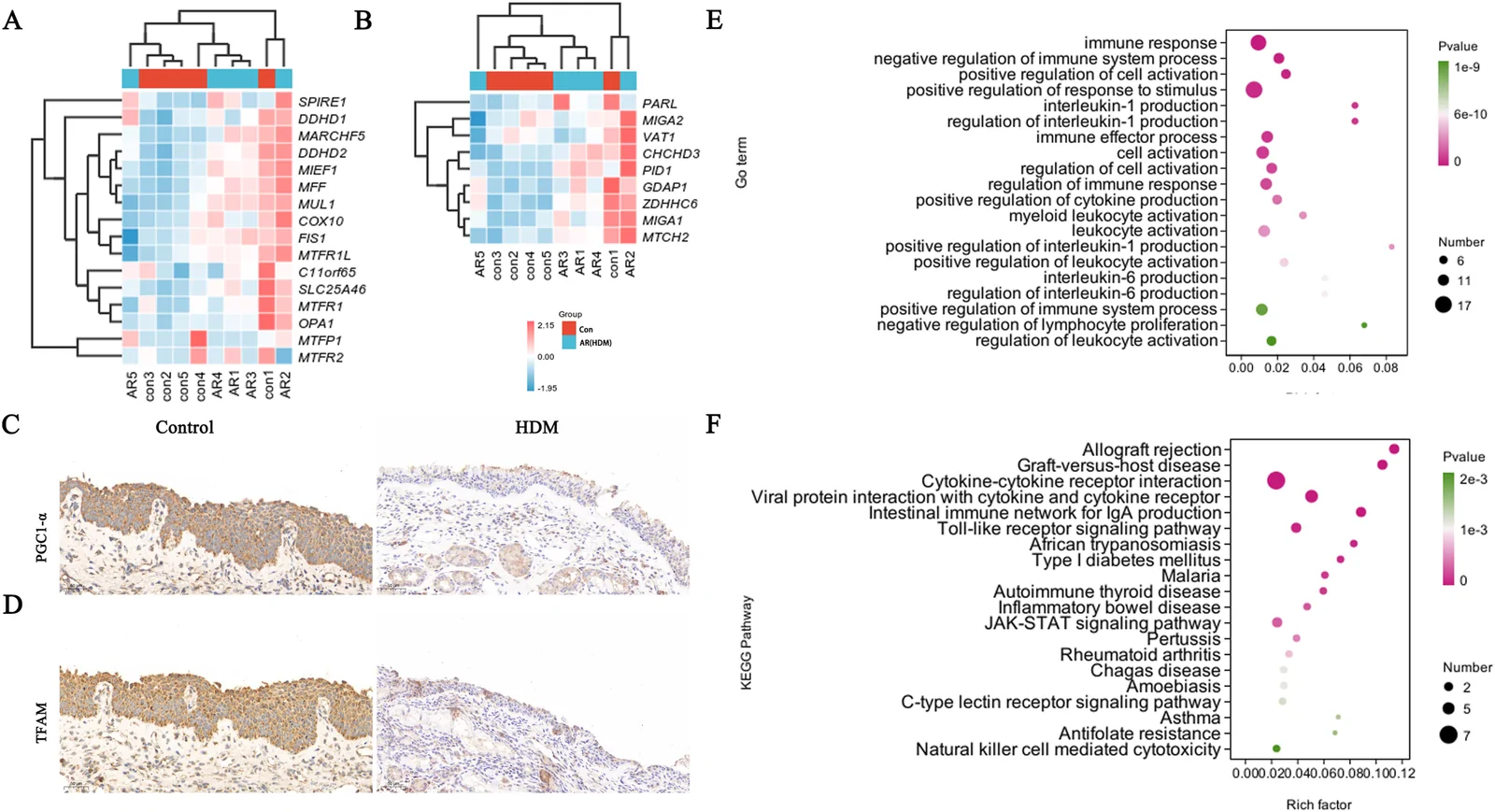
RNA-seq analysis of nasal mucosa of the two groups (Control, AR). (A-B) Heatmap of the differentially expressed gene generated from the nasal mucosa RNA-seq of AR vs Control groups: (A) Heatmap of genes related to mitochondrial fission B) Heatmap of genes related to mitochondrial fusion (C-D) Immunohistochemical images of PGC-1α and TFAM expression in nasal mucosa of AR vs Control groups (E) The dot plot of GO-BP, CC, MF annotation enrichment of differentially expressed genes in different groups. (F) Dot plot of KEGG pathway enrichment analyses of differentially expressed genes in different groups.

We collected the nasal mucosa from both AR patient and health control, part of the mucosa was used for immunochemistry experiment, and the other part was used for RNA-seq analysis. In the heatmap, the region from blue to red represents the increasing abundance of differentially expressed genes (DEGs) (cut off 2-fold change; adjusted P < 0.05; Fig. 1A–B). In most AR groups, the relevant genes which related with mitochondrial fission and fusion are upregulated (Fig. 1A–B). By contrast, these genes downregulated in control group. According to the immunohistochemical results in Fig. 1C, D, the expression of mitochondrial biogenesis related proteins PGC-1α and TFAM in the nasal mucosal epithelium of AR patient was lower than that in the normal control group.

As presented in Fig. 1E, Gene Ontology (GO) terms of most DEGs were primarily involved in interleukin-1 production, regulation of interleukin-1 production, interleukin-6 production, regulation of interleukin-6 production, positive regulation of immune system process and immune response in the AR group compared to the control group. Subsequently, KEGG pathway enrichment analysis of DEGs is comparing the control and AR groups showed that these DEGs were mainly linked to the JAK-STAT signaling pathway, asthma, cytokine-cytokine receptor interaction (Fig. 1F). The above results summarize that, compared with the control group, mitochondrial function in the AR group is significantly more active and metabolic levels are increased. At the same time, oxidative stress and phosphorylation pathways are activated, and interleukin-1 and interleukin-6 also play important roles in the complex inflammatory response. Therefore, we have focused our research on the functions of mitochondria and the roles of interleukin-6 and interleukin-1, aiming to inhibit the progression of AR inflammation and achieve therapeutic effects by regulating mitochondrial function, oxidative stress responses, and suppressing changes in inflammatory factors.

### 3.2 Fabrication, characterization and properties of apt-BUD/C@MnO_2_ NFs

**Fig. 2:**
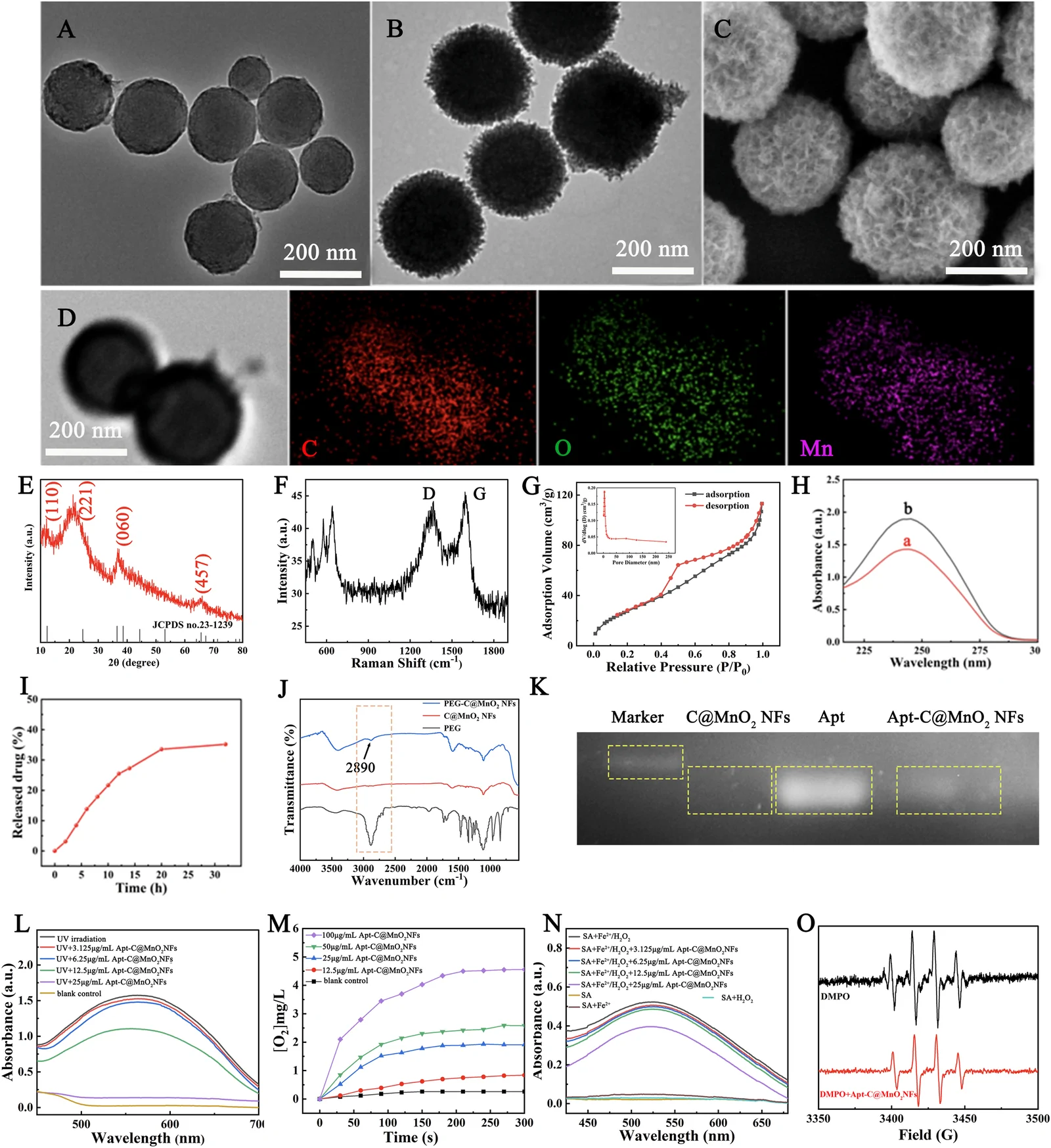
TEM images of (A) PCNSs, (B) C@MnO_2_ NFs. SEM images of (C) C@MnO_2_ NFs. (D) HRTEM images and element mapping of C@MnO_2_ NFs. (E) XRD spectrum of C@MnO_2_ NFs. (F) Raman spectrum of C@MnO_2_ NFs. (G) N_2_ adsorption-desorption isotherms and pore size distributions (inset image) ofC@MnO_2_ NFs. (H) UV-Vis spectra of BUD solution before and after mixing with apt-C@MnO_2_ NFs. (I) BUD release from Apt-C@MnO_2_NFs loaded with BUD at pH = 7.4. (J) FTIR spectra of C@MnO_2_ NFs before and after PEG modification. (K) Gel electrophoresis image of C@MnO_2_ NFs before and after modification of Apt. (L) Scavenging of O_2_^.−^by Apt-C@MnO_2_ NFs with different concentrations. (M) Produced oxygen curve of Apt-C@MnO_2_ NFs mixed with H_2_O_2_. (N) Scavenging of·OH by Apt-C@MnO_2_ NFs with different concentrations. (O) ESR spectra of·OH scavenging by DMPO capturing Fenton reaction system.

**Fig. 3:**
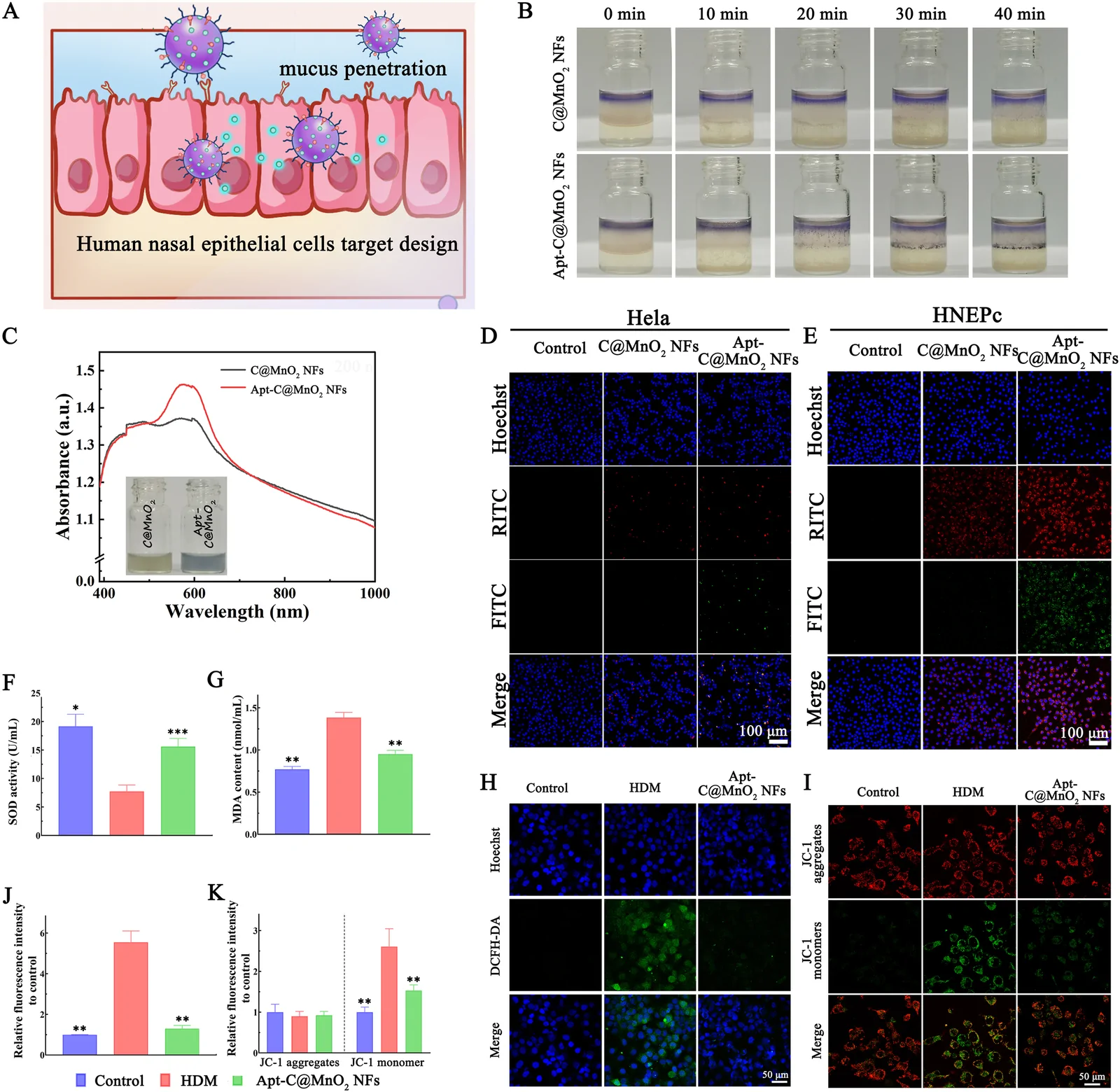
(A) Scheme illustration of mucus penetration and HNEPc targeting. (B) Simulating the penetration of artificial mucus layer. (C) UV-Vis absorption spectra of gelatin layer penetration of coomassie brilliant blue labeled C@MnO_2_ NFs and Apt-C@MnO_2_ NFs. Cell targeting experiment: The CLSM images of RITC labeled C@MnO_2_ NFs before and after modification of FITC labeled Apt to enter (D) Hela cells and (E) HNEPc, respectively. (F) SOD and (G) MDA level of HNEPc treated with PBS (control), HDM and HDM + Apt-C@MnO_2_. The CLSM images of HNEPc stained with (H) DCFH-DA and (I) JC-1 after treated with PBS (control), HDM or HDM + Apt-C@MnO_2_ NFs. (J) Mean fluorescent intensity corresponding to (H). (K) Semi quantitative analysis of JC-1 aggregates and JC-1 monomers fluorescence intensity corresponding to (I). (For interpretation of the references to color in this figure legend, the reader is referred to the Web version of this article.)

The PAA-Zn NPs were prepared by adding an appropriate amount of ZnO to the PAA aqueous solution and then adding isopropanol dropwise after ultrasonic stirring for a certain period of time. The dried PAA-Zn NPs were calcined in an argon furnace at 900 °C for 3 h. During this process, PAA was carbonized and Zn^2+^ was burned off at high temperatures to form porous carbon spheres (PCNSs). As shown in Fig. 2A, transmission electron microscopy (TEM) images showed that the PCNSs with good dispensability, uniform size, smooth surface, and a size of approximately 180 ± 10 nm. Owing to the reducibility of carbon and the oxidability of KMnO_4_, MnO_2_nanoflowers were formed on the surface of PCNSs under heating to obtain C@MnO_2_ NFs. As shown in Fig. 2B–C, TEM and scanning electron microscopy (SEM) images showed that the size of the synthesized C@MnO_2_ NFs was larger than that of PCNSs, about 220 ± 10 nm, and the surface changed fromsmooth to flower-like nanospheres composed of flake-like structures with uniform distribution of flakes.Fig. 3D was a high-resolution transmission electron microscope (HRTEM) image of C@MnO_2_ NFs and its corresponding elemental mapping of C, O, and Mn elements. It can be seen that the C element was mainly concentrated in the central position, and the O and Mn elements were mainly distributed in the outer layer, indicating the C@MnO_2_ NFs were successfully synthesized. Then, we carried out XRD tests (Fig. 2E), confirming the presence of MnO_2_ by comparison with the JCPDS 23-1239 standard card of MnO_2_. Fig. 2F shows that the C@MnO_2_ NFs have two significant peaks at 1357 cm^−1^ and 1600 cm^−1^, which are attributed to the disordered D and G bands of carbon, respectively. There are also three peaks at 502 cm^−1^, 577 cm^−1^ and 650 cm^−1^, which are well matched with the characteristic peaks of MnO_2_. The Raman spectroscopy results proved that C and MnO_2_ coexisted, which proved that the C@MnO_2_ NFs were successfully prepared. The MnO_2_content was determined by inductively coupled plasma atomic emission spectrometry (ICP-AES). The results show that the MnO_2_ was approximately 20.9 wt %. We further studied the electronic structure and chemical composition of the C@MnO_2_ NFs in detail. The results of X-ray photoelectron spectroscopy (XPS) were shown in Fig. S1. The characteristic peak of C 1s at 285 eV, O 1s at 531.3 eV and 529.5 eV confirmed that the C@MnO_2_ NFs contained C and O element. The XPS binding energies of Mn were 642 eV and 653.8 eV, respectively, which are related to Mn 2p_3/2_ and Mn 2p_1/2_.

The nitrogen adsorption desorption curve in Fig. 2G shows that it is a type IV isotherm and an H3 hysteresis loop, which can be considered as a slit pore formed by the accumulation of flake particles. The pore size was 4 nm, and the BET surface area was 110 m^2^/g, which has mesopores and a large specific surface area. Benefit from this property, Apt-C@MnO_2_ NFs can load drugs to form a synergistic inflammatory treatment system. From Fig. 3H, a decrease in absorbance before and after BUD loading was observed, which proved that Apt-C@MnO_2_ NFs could successfully load drugs, and the drug loading rate was 19.8 %. By adjusting the initial drug amount, we could control drug loading, achieve the synergistic effect of nanozymes and drugs, and provide a basis for personalized clinical treatment. Additionally, the release behavior of BUD in PBS was analyzed. Fig. 3I shows that the initial release rate of BUD was fast and then gradually slowed, indicating that Apt-C@MnO_2_ NFs can achieve sustained release of drugs, extend the period of effectiveness, and improve the anti-inflammatory therapeutic outcome.

EpCAM aptamer (Apt) was chosen to target epithelial cell adhesion molecule, which is highly expressed on the surface of epithelial cells. Before conjugation with the Apt, poly (ethylene glycol) 2-aminoethyl ether acetic acid ((NH_2_-PEG-COOH) was modified on the surface of MnO_2_ to facilitate their penetration through the nasal mucus. Fourier transform infrared spectroscopy showed that the PEG-C@MnO_2_ NFs spectrum contained the characteristic absorption peak of -CH_3_(2890 cm^−1^), which proved the successful modification of PEG on the C@MnO_2_ NFs surface (Fig. 2J). Then, the Apt with an amino group was successfully connected to the carboxyl group of PEG on the surface of the C@MnO_2_ NFs to form Apt-C@MnO_2_ NFs. As shown in Fig. 3K, the gel electrophoresis image shows that the Apt band was bright, and there is no band in the single nanomaterial, whereas the remaining Apt band was significantly lighter after the formation of the composite material Apt-C@MnO_2_ NFs, indicating that the Apt was successfully connected, which proved the successful preparation of Apt-C@MnO_2_ NFs. In addition, the appearance of a characteristic ∼260 nm absorption peak in UV-vis spectra and the obvious positive shift of zeta potential jointly confirm successful aptamer modification onto C@MnO_2_ nanoflowers (Fig. S2 and S3). Fig. S4 demonstrates that no notable alterations were evident in the morphology of the particles after the modification of the Apt, indicating that the modification did not change the particle structure.

After the successful synthesis of the Apt-C@MnO_2_ NFs, their scavenging effects on different types of reactive oxygen species (·OH, O_2_^.−^and H_2_O_2_) were verified. It can be seen that after adding Apt-C@MnO_2_ NFs, the characteristic absorption peak of the mixed solution (riboflavin, methionine and NBT) decreased significantly at 560 nm, which proved its ability to remove O_2_^.-^ in Fig. 2L. In addition, Apt-C@MnO_2_ NFs could effectively catalyze the conversion of H_2_O_2_ to O_2_, resulting in an increase in the O_2_ concentration in the solution (Fig. 2M), confirming the CAT activity of Apt-C@MnO_2_ NFs. When the concentration of Apt-C@MnO_2_ NFs was increased, the oxygen production increased faster and more simultaneously, indicating their concentration dependent character. From Fig. 2N, we observed that the Apt-C@MnO_2_ NFs could scavenge ·OH, and the scavenging rate of ·OH was 24.4 % at the particle concentration of 25 μg/mL. Finally, we used ESR spectroscopy combined with spin trapping technology to detect the scavenging ability of the Apt-C@MnO_2_ NFs for ·OH. The DMPO reaction can capture the transiently highly active ·OH radical formation characteristic spectrum. In the sample containing only DMPO, an obvious four-line spectrum with a relative intensity of 1: 2: 2: 1 was detected as an ESR signal, which is the characteristic spectrum of DMPO/·OH (Fig. 2O). In contrast, in the presence of Apt-C@MnO_2_ NFs, the ESR signal was significantly weakened, further demonstrating the ·OH scavenging ability of the Apt-C@MnO_2_ NFs.

### 3.3 Mucus penetration and HNEPc targeting abtiliy of Apt-C@MnO_2_ NFs

For AR treatment, faster mucus penetration and HNEPc targeting may induce more effective treatment outcome (Fig. 3A). The presence of PEG in the outer layer of Apt-C@MnO_2_ NFs can effectively prevent its interaction with mucus, so that the Apt-C@MnO_2_ NFs did not easily adhere when they came into contact with mucus, which effectively reduced non-specific adhesion and improved the free diffusion ability of NFs in mucus. As shown in Fig. 3B, it can be observed that both C@MnO_2_ NFs and Apt-C@MnO_2_ NFs could enter the mucus layer over time, and the penetration rate of Apt-C@MnO_2_ NFs was significantly greater than that of C@MnO_2_ NFs. By analyzing the absorbance of the agarose gel layer after penetration for 40 min, the penetration ability of the mucus was quantitatively determined (Fig. 3C). In summary, Apt-C@MnO_2_ NFs exhibited better mucus permeability than the unmodified C@MnO_2_ NFs.

To explore the targeting ability of Apt-C@MnO_2_ NFs to HNEPc, RITC labeled C@MnO_2_ NFs, before and after modification of FITC labeled Apt were added to HeLa cells and HNEPc for 3 h under the same conditions. Then, the distribution of NFs in the cells was observed using CLSM. Since theApt was only secreted in HNEPc and not in.

Hela cells, CLSM imaging showed that less Apt-C@MnO_2_ NFs were phagocytized by HeLa cells in 3h (Fig. 3D), but they could target HNEPc more efficient (Fig. 3E).

Moreover, compared with Apt-C@MnO_2_ NFs, red fluorescence was also observed in HNEPc treated with C@MnO_2_ NFs, but it was obviously less than that of Apt-C@MnO_2_ NFs (Fig. 3E). The results showed that Apt-C@MnO_2_ NFs have the ability to target HNEPc, which may greatly improve the AR therapeutic effect.

### 3.4 ROS elimination and mitochondrial function recovery via Apt-C@MnO_2_ NFs

Before testing the therapeutic effect of the Apt-C@MnO_2_ NFs, the cytotoxicity of the HNEPc was analyzed using the MTT method. As shown in Fig. S5, the survival rate of HNEPc was greater than 85 % after treatment with Apt-C@MnO_2_ NFs at a concentration of 25 μg/mL for 24 h. The above experimental results show that HNEPc haves good biocompatibility, making them suitable possible for further biological applications. In the following experiments, all the HNEPc were stimulated by HDM except the control group. We first test the SOD and MDA level after treated with pure HDM and HDM + Apt-C@MnO_2_ NFs. The data in Fig. 3F show that the SOD activity of the control group stimulated by HDM decreased to 7.73 U/mL, while the SOD activity of the group treated with Apt-C@MnO_2_ NFs increased back to 14.60 U/mL. MDA results (Fig. 3G) showed that HDM stimulation led to an increase in MDA content in the control group, whereas after Apt-C@MnO_2_ NFs treatment, oxidative stress was reduced, ROS attack on cells was reduced, and oxidative damage was alleviated. Then, the ability of the Apt-C@MnO_2_ NFs to clear ROS *in vitro* has been validated in Fig. 3H and J. Using DCFH-DA as a fluorescent probe to detect ROS content in cells, the stronger the green fluorescence, the higher the ROS content in the cells. After incubating the HNEPc for 24 h, the HDM stimulating solution was added to generate ROS in the CLSM images. No green fluorescence was observed in the control group, suggesting a low level of ROS in these samples. The fluorescence intensity of the HDM + Apt-C@MnO_2_ NFs group decreased significantly, which indicated that the Apt-C@MnO_2_ NFs had a good ability to scavenge ROS in cells. These results indicate that the Apt-C@MnO_2_ NFs can alleviate ROS in HNEPc.

Mito-tracker was used to stain mitochondria, verify whether Apt-C@MnO_2_NFs can enter the mitochondria and have a certain impact on mitochondrial function. As shown in Fig. S6, it can be seen that the green fluorescence in HNEPc treated with HDM was weakened, indicating impaired mitochondrial function. This may be because the stimulation of HNEPc by allergens inhibits mitochondrial biogenesis. After Apt-C@MnO_2_ NFs treatment, the red fluorescence of the Apt-C@MnO_2_ NFs was uniformly distributed in the cells, and the green fluorescence intensity returned to normal levels. The overlap between red fluorescence and green fluorescence indicates that Apt-C@MnO_2_ NFs can enter the mitochondria, while stronger green fluorescence indicates the restoration of mitochondrial function. In addition, mitochondrial membrane potential testing was used to detect mitochondrial damage, and JC-1 served as a fluorescent probe to determine the level of membrane potential. When the probe changed from red to green fluorescence, it represented a decrease in the membrane potential and was also a marker of mitochondrial damage. As shown in Fig. 3I and K, green fluorescence was enhanced in HNEPc treated with the HDM stimulating solution, indicating impaired mitochondrial function. Therefore, after the addition of Apt-C@MnO_2_ NFs, the green fluorescence decreased and the red fluorescence recovered, indicating that NFs can restore mitochondrial function.

To explore the energy metabolism of HNEPc in the anti-inflammatory process of Apt-C@MnO_2_ NFs, ATP levels in HNEPc treated under different conditions were studied. As shown in Fig. S7, the ATP level produced in HNEPc treated with HDM was significantly reduced, indicating that HDM, an allergen, inhibited mitochondrial biogenesis, impaired mitochondrial function, and significantly inhibited ATP production in cells. The HNEPc, which is stimulated by HDM, then were treated with BUD, C@MnO_2_ NFs, BUD/C@MnO_2_ NFs and BUD/Apt-C@MnO_2_ NFs, ATP levels increased to varying degrees, indicating recovery of mitochondrial function. Among them, the Apt-BUD/C@MnO_2_ NFs had the most significant recovery effect, almost similar to that of normal HNEPc. This result lays the foundation for the application of Apt-BUD/C@MnO_2_ NFs *in vivo*.

### 3.5 Apt-BUD/C@MnO_2_ NFs suppress inflammatory response in AR and JAK-STAT, P13K-Akt pathway is involved

**Fig. 4:**
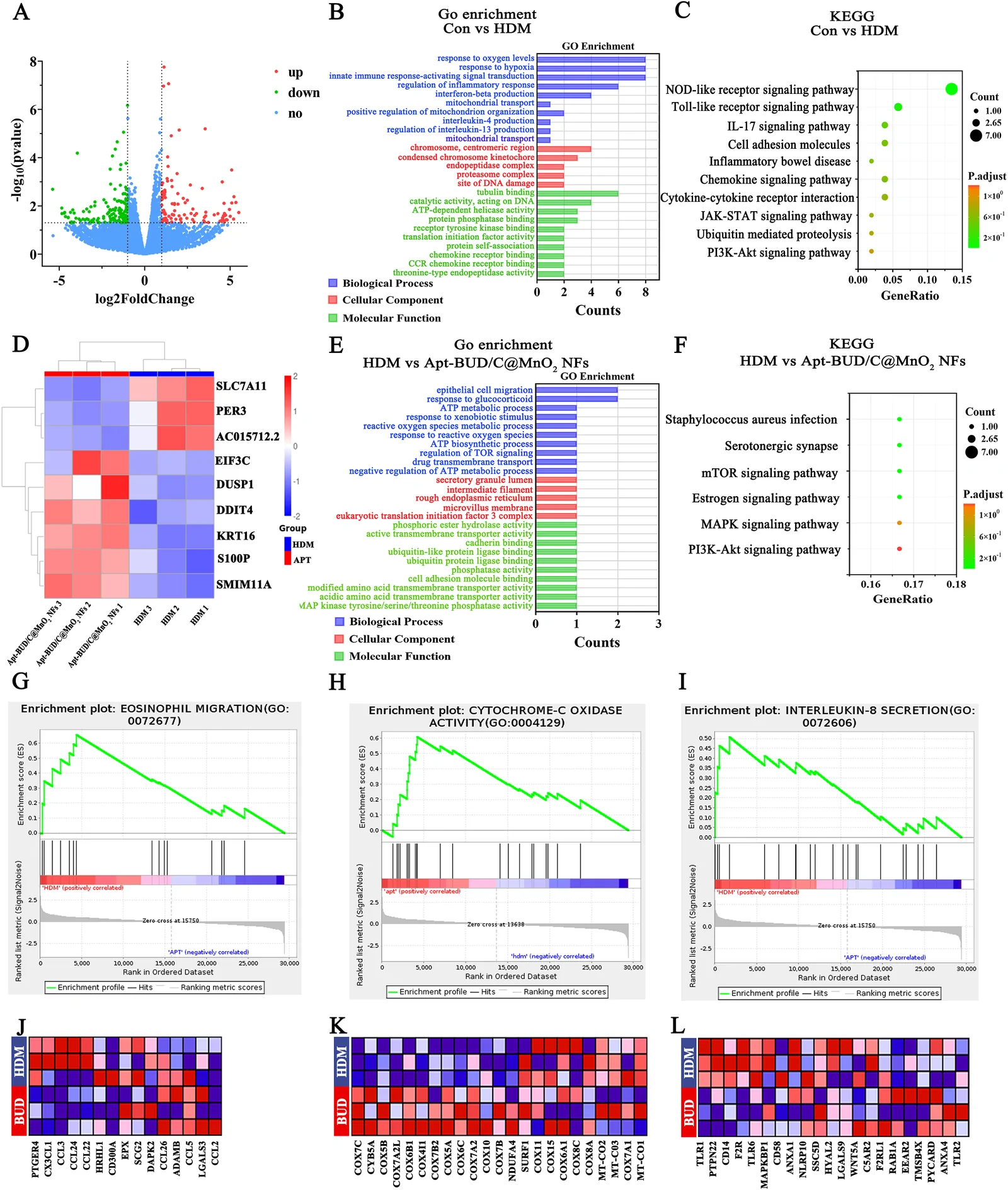
RNA-seqanalysisof HNEPc in the three groups (Control, HDM, HDM + Apt-BUD/C@MnO_2_ NFs groups). (A) Differential gene expression visualized through a volcano plot (HDM vs HDM + Apt-BUD/C@MnO_2_ NFs groups). Upregulated genes are depicted in red, while downregulated genes are shown in green. (B) and (E) The bar chart of GO-BP, CC, MF annotation enrichment of differentially expressed genes in different groups (Control, HDM, HDM + Apt-BUD/C@MnO_2_ NFs groups). (C) and (F) Dot plot of KEGG pathway enrichment analyses of differentially expressed genes in different groups (Control, HDM, HDM + Apt-BUD/C@MnO_2_ NFs groups). (D) Heatmap of the differentially expressed gene generated from the HNEPc RNA-seq of HDM vs Control groups. (G-J) GSEA and heatmap of eosinophil migration in different groups. (H-K) GSEA and heatmap of cytochrome-c oxidase activity in different groups. (I-L) GSEA and heatmap of interleukin-8 secretion in different groups.P-values are represented on a gradient (from red to green). The size of the bubbles reflects the degree of enrichment. BP,Biological Process; CC, Cellular Component; MF, Molecular Function, KEGG, Kyoto Encyclopedia of Genes and Genomes.Apt-BUD/C@MnO_2_ NFs is used in the figure legend to refer to the HDM + Apt-C@MnO_2_ NFs group. (For interpretation of the references to color in this figure legend, the reader is referred to the Web version of this article.)

To identify the molecular changes in the nasal epithelial of control, HDM and HDM + Apt-BUD/C@MnO_2_ NFs groups, we performed whole-transcriptome gene expression analysis. The transcriptome assay enabled the detection of the differential expression of 956 transcripts. Volcano plots of transcriptome sequencing data were used to visualize the distribution of DEGs between the two groups (Fig. 4A). In the heatmap, the region from blue to red represents the increasing abundance of DEGs (cut off 2-fold change; adjusted P < 0.05; Fig. 4D). As presented in Fig. 4B, GO terms of most DEGs were primarily involved in Th2 cytokines and other related inflammatory factors in the HDM group compared to the control group: regulation of interleukin-13 production, interleukin-4 production, regulation of inflammatory response, interferon-beta production, protein phosphatase binding, receptor tyrosine kinase binding, and CCR chemokine receptor binding. The GO terms of most DEGs (Fig. 4E) were related to mitochondrial and oxidative stress: including response to oxygen levels, response to hypoxia, mitochondrial transport, positive regulation of mitochondrion organization, mitochondrial transport and ATP-dependent helicase activity has also been displayed. Subsequently, KEGG pathway enrichment analysis of DEGs comparing the control and HDM groups showed that these DEGs were mainly linked to the NOD-like receptor signaling pathway, Toll-like receptor signaling pathway, IL-17 signaling pathways, including the chemokine signaling pathway, cytokine–cytokine receptor interaction, JAK-STAT signaling pathway, and PI3K-Akt signaling pathway (Fig. 4C). Analysis of Gene Ontology annotations and KEGG pathway enrichment for the HDM and Apt-BUD/C@MnO_2_ NFs groups revealed that the DEGs were primarily linked to the IL-17 signaling pathway and its relation pathways: JAK-STAT signaling pathway, and P13K-Akt signaling pathway, mTOR signaling pathway, estrogen signaling pathway and MAPK signaling pathway revealed that TH17 cells play a vital role in HDM allergic reactions (Fig. 4F). GSEA and heatmap illustrated that CCL5, which plays a key role in the pathological process of AR by driving eosinophil infiltration and maintaining the Th2 inflammatory milieu, downregulated in Apt-BUD/C@MnO_2_ NFs groups compared with HDM groups (Fig. 4G). In our research,we also found CCL-3,CCL22 and CCL-24 was the most prominent eotaxin which attract eosinophil in HDM induced allergic rhinitis model.Previous studies have reported that eosinophils in blood circulation will be attract by eotaxin(such as CCL-11,CCL24,CCL26) transmigrate across vascular endothelium and infiltrate into the airway mucosa [20,31]. It was reported that CCL24 had a greater influence on eosinophil recruitment into the bronchoalveolar lavage in than CCL11 in ovalbumin (OVA)-induced asthma model. CX3CL1 was upregulation in HDM group compared with Apt-BUD/C@MnO_2_ NFs groups in our research(Fig. 4J). In previous study, it was noted that CX3CL1 [32]can attenuate lung fibroblast activation and mitochondrial dysfunction, which leads to the tissue remodeling in ovalbumin-challenged mice model. Thus according to our result, the Apt-BUD/C@MnO_2_ NFs possesses a certain capacity to inhibit epithelial mucosal tissue remodeling.Thus we can conclude the Apt-BUD/C@MnO_2_ NFs can inhibit the recruitment of eosinophils from blood and their transendothelial migration across vascular endothelium into nasal mucosal tissue, thereby alleviating tissue remodeling induced by inflammatory cell infiltration. COX subunit genes (e.g., COX7C, COX5B, COX7A2L et al.) deficiency can lead to mitochondrial complex dysfunction, resulting in reduced ATP synthesis and excessive ROS accumulation. The COX subunit genes were upregulated in Apt-BUD/C@MnO_2_ NFs groups compared with HDM groups (Fig. 4H–K).TLR1、PTPN22、CD14、F2R and IL-8 were upregulated in Apt-BUD/C@MnO_2_ NFs groups compared with HDM groups(Fig. 4I–L), which implies that inflammation is polarized in the TH1 direction rather than the TH2 direction.

As mentioned above, eosinophil infiltration was prominent in the epithelial mucosa of the HDM group. To investigate the effects of eosinophils on epithelial cells, we evaluated epithelial alterations from four perspectives: epithelial barrier integrity, epithelial inflammatory response, epithelial injury, and epithelial remodeling (Fig.S12 A-D), with comparisons made against the Apt-C@MnO_2_ NFs treatment group. Combined with the analysis results of GO biological process (Fig.S12 G) enrichment and the PPI network(Fig.S12 F) indicated that HDM-triggered chemokine cascades facilitate robust eosinophil recruitment to nasal mucosa; subsequently, inflammatory mediators and matrix metalloproteinases disrupt tight junction assembly, leading to increased epithelial permeability. Apt-C@MnO_2_ NFs treatment alleviated the severe inflammation caused by eosinophil, reversed the nasal epithelial barrier injury and tissue remodeling, which evealed its promising prospects as a therapeutic agent against allergic rhinitis.

Our data revealed how HDM sensitize the complex inflammatory process of HNEPc and how Apt-BUD/C@MnO_2_ NFs intervenes in this process at the mRNA level. Molecular function category GO terms associated with the majority of DEGs between HDM and Apt-BUD/C@MnO_2_ NFs were primarily involved in ATP metabolic processes, ATP biosynthetic processes, and negative regulation of ATP metabolic processes, which illustrated that oxidative damage was alleviated.

**Fig. 5:**
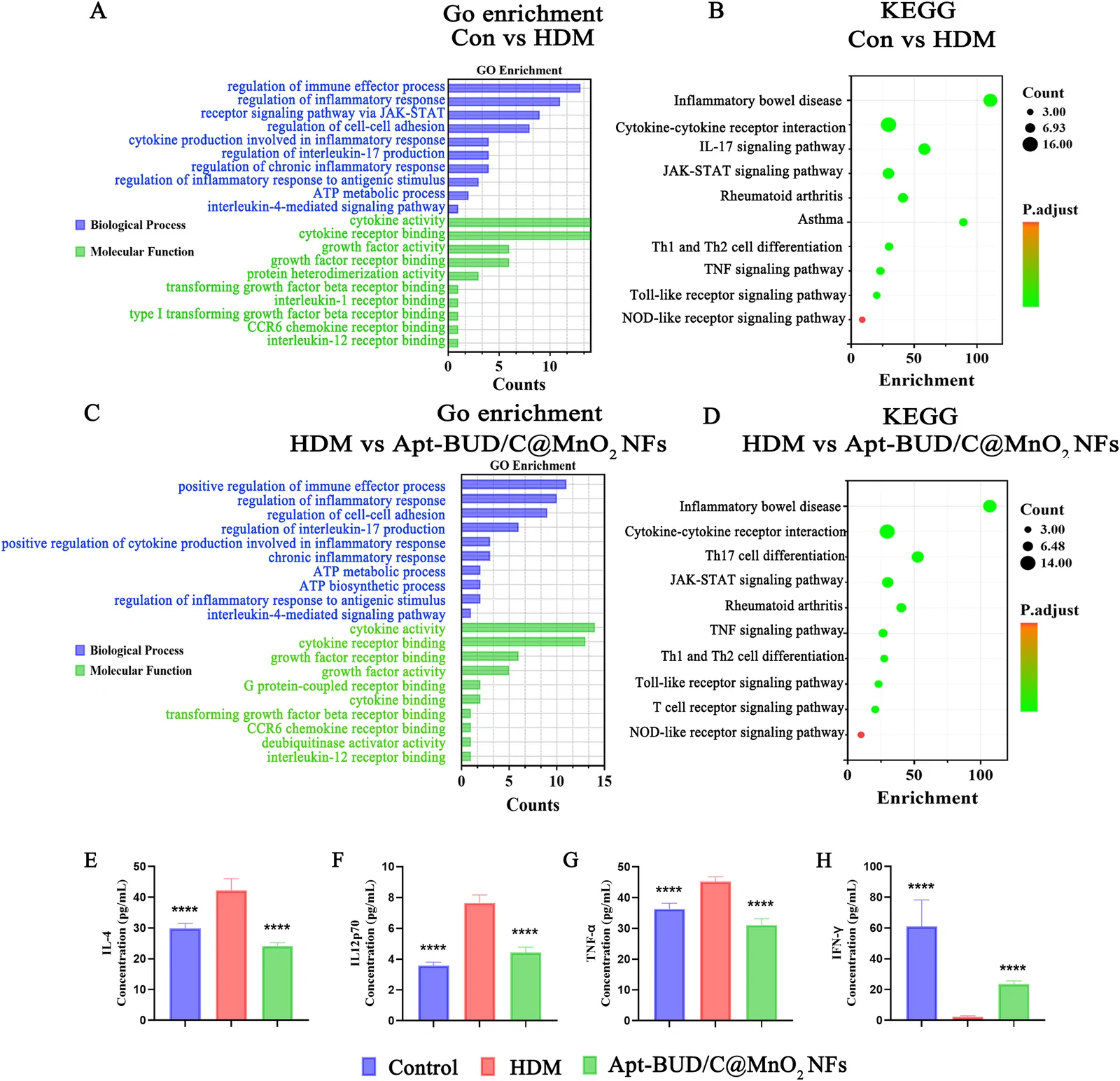
Enrichment of differential expressed genes in the cytokine antibody array. GO (A), (C) and KEGG (B), (D) enrichment analyses of differentially expressed cytokine genes detected in HNEPc of the three group (Control, HDM, Apt-BUD/C@MnO_2_ NFs). (E-H) Effects of HDM and Apt-BUD/C@MnO_2_ NFs on cytokine secretion in HNEPc. The relative expression levels of (E)IL-4, (F)IL-12P70, (G) TNF-α, (H)IFN-γ were assayed using antibody arrays. Data are expressed as mean ± SEM. Group differences were evaluated using ANOVA.Apt-C@MnO_2_ NFs is used in the figure legend to refer to the HDM + Apt-C@MnO_2_ NFs group.

**Fig. 6:**
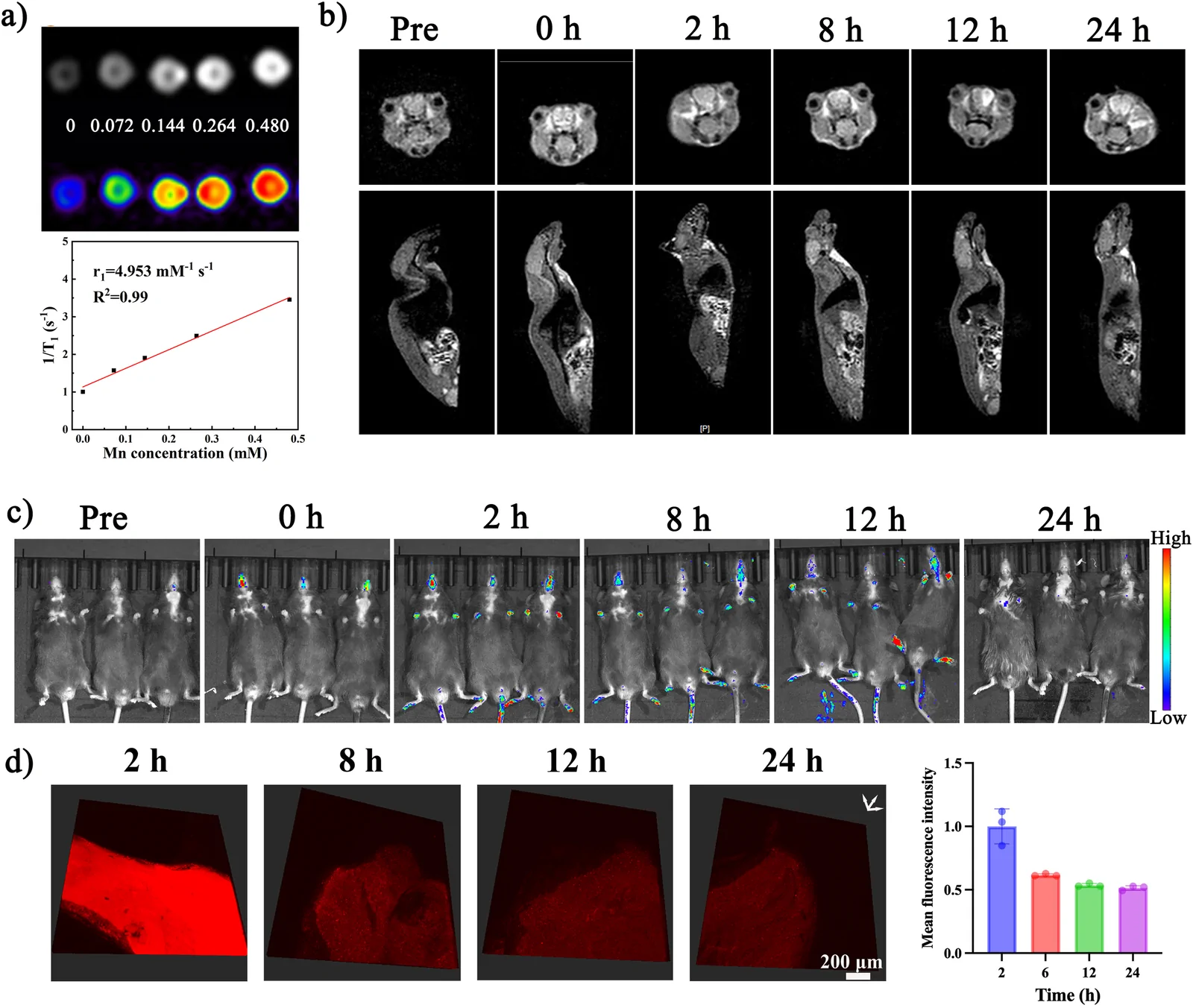
(A) MRI images of different concentrations of Apt-C@MnO_2_ NFs solution and linear fitting diagram of the corresponding relationship between 1/T1 and Mn element concentration. (B) MRI images and (C) Fluorescence images in mice before and after nasal administration of Apt-C@MnO_2_ NFs. (D) 3D CLSM images of nasal tissue after intranasal treatment to qualitatively verify the *in vivo* mucosal penetration of RhB-Apt-C@MnO_.2_ NFs, and corresponding mean fluorescence intensity.

GO analysis of differentially expressed genes in the antibody array in HNEPc for the control, HDM and HDM + Apt-BUD/C@MnO_2_ NFs groups (Fig. 5A–C). As shown in Fig. 6A, in control and HDM groups, GO terms of most DEGs were primarily involved in the regulation of the immune effector process, regulation of inflammatory response, receptor signaling pathway via JAK-STAT, regulation of interleukin-17 production, CCR6 chemokine receptor binding, interleukin-12 receptor binding, interleukin-4-mediated signaling pathway, and ATP metabolic process. In the HDM and HDM + Apt-BUD/C@MnO_2_ NFs groups (Fig. 5C), GO terms of most DEGs were primarily involved in regulation of interleukin-17 production, interleukin-4-mediated signaling pathway, CCR6 chemokine receptor binding, and interleukin-12 receptor binding, regulation of inflammatory response, regulation of cell-cell adhesion, ATP metabolic process, and ATP biosynthetic process. As presented in Fig. 5B–D, KEGG pathway enrichment analysis of the control, HDM and HDM + Apt-BUD/C@MnO_2_ NFs.

Groups illustrated that the DEGs were primarily linked to the IL-17 signaling pathway, JAK-STAT signaling pathway, Th1 and Th2 cell differentiation, and T cell receptor signaling.

Cytokines were compared with the antibody array in HNEPc among the control, HDM and Apt-BUD/C@MnO_2_ NFs groups. The levels of pro-inflammatory cytokines (TNF-α and IL12p70) and Th2 related cytokines (IL-4) were decreased but Th1 related cytokines (IFN-γ) were increased in the Apt-BUD/C@MnO_2_ NFs group when compared to those in the HDM group (Fig. 5E–H).

### 3.6 *In vivo* image guided anti-AR effect: symptom, cilia damage, eosinophil infiltration, mitochondrial biogenesis and epithelial remodeling

Before testing the *in vivo* anti-AR effect, we examined the effects of different concentrations of Apt-C@MnO_2_ NFs on the hemolysis of red blood cells (Fig. S8). The hemolysis of red blood cells was almost not observed in the concentration range of 12.5-400 μg/mL, and the hemolysis rate of red blood cells was also lower than 5.0 % at a concentration of 400 μg/mL, which indicating that the Apt-C@MnO_2_ NFs have good biocompatibility and can be used for biological *in vivo* experiments. We then investigated the imaging effect of Apt-C@MnO_2_ NFs. MnO_2_ has been shown to be a contrast agent for T1-weighted MRI. Fig. 6A shows T1-weighted MRI images of the Apt-C@MnO_2_ NFs solutions containing different concentrations of Mn^2+^. There was a good linear relationship between the 1/T1 value and the concentration of Mn, and the slope of this line was the relaxation rate (R1, 4.953 mM^−1^ s^−1^). As shown in Fig. 6B, Apt-C@MnO_2_ NFs in mice gradually enriched in the nose with time and gradually decreased with time after 8 h. Meanwhile, fluorescent imaging has the advantages of high selectivity, high sensitivity, and non-invasive real-time imaging. The prepared Apt-C@MnO_2_ NFs loaded.

With fluorescent markers (RITC) was tested in mice (Fig. 6C). The results showed that the RITC labeled Apt-C@MnO_2_ NFs retained a relatively high level at 12 h and almost disappeared at 24 h, indicating that these materials can be used as long-term treatment agents and cleared after treatment. Furthermore, time-dependent 3D CLSM imaging of nasal tissue *in vivo* demonstrated that the nanoparticles gradually penetrated through the nasal mucus layer and diffused into deep nasal tissue within 24 h after intranasal administration in Fig. 6D, which further verified its mucus-penetrating property.

**Fig. 7:**
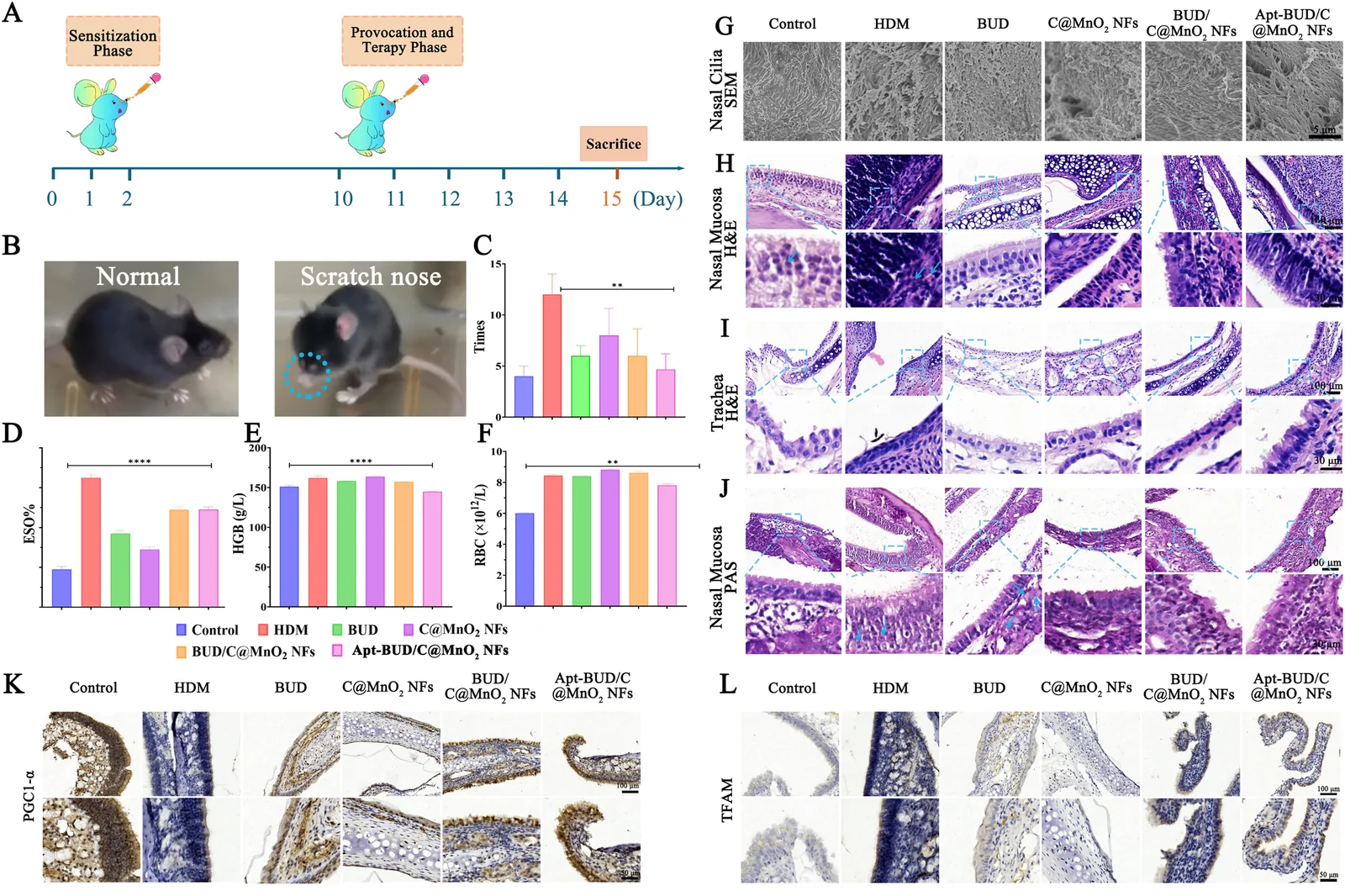
(A) Establishment of mouse model and treatment process. (B) Symptom photos of AR mice: scratch the nose and sneeze. (C) Show the frequency of symptoms in different groups of mice. Statistical significance: *p < 0.05. Blood routine of mice (D) percentage of eosinophils (ESO%); (E) hemoglobin (HGB); (F) red blood cells (RBC). (G) SEM images of mice: cilium of nasal epithelium. (H) Hematoxylin and eosin(H&E) staining of nasal mucosa of mice (magnification, ×100 Bar indicates 100 μm, ×400 Bar indicates 30 μm) The blue arrows indicate the identified eosinophils in the respiratory epithelium. (I) H&E staining of trachea of mice (magnification, ×100 Bar indicates 100 μm, ×400 Bar indicates 30 μm). (J) Periodic Acid-Schiff (PAS) staining of nasal mucosa of mice (magnification, ×100 Bar indicates 100 μm, ×400 Bar indicates 30 μm) The blue arrows indicate the identified eosinophils goblet cells in the respiratory epithelium. (K, L) Immunohistochemical images of PGC-1α and TFAM expression in nasal mucosa of mice from each group. Apt-C@MnO_2_ NFs is used in the figure legend to refer to the HDM + Apt-C@MnO_2_ NFs group. (For interpretation of the references to color in this figure legend, the reader is referred to the Web version of this article.)

To estimate the AR therapeutic effect of Apt-BUD/C@MnO_2_ NFs, a mouse AR model was successfully established using the HDM as stimulant. After inflammation occurred in the nose of the mice, we administered nasal dropsof different materials to the randomly assigned mice for 5 days (Fig. 7A). After sensitization, the mice exhibited symptoms such as scratching their noses and sneezing (Fig. 7B). By observing and comparing the frequency of AR symptoms in mice (Fig. 7C), it can be seen that the mice treated with the HDM + Apt-BUD/C@MnO_2_ NFs had the most significant symptom reduction. This indicates that our nanozyme drug nanosystemis effective in the treatment of AR. Routine blood tests and histological analyses of the major organs were performed. As shown in Fig. 7D, the percentage of eosinophil in AR mice was significantly reduced after treatment, whereas hemoglobin and red blood cell counts (Fig. 7E and F) were not significantly changed.

By comparing the pathological changes in the nasal cilia, nasal mucosa and trachea of mice in each group (Fig. 7G–I), it was observed that the normal nasal mucosal tissue of mice had regular, dense cilia, uniform staining, and a complete epithelial structure of the mucosal layer. The epithelium was a pseudostratified ciliated columnar epithelium, with normal morphology and structure of HNEPc and no obvious inflammation. The arrangement of cilia in the nasal mucosa of mice stimulated by HDM was disordered, with some cilia sticking to clusters and tilting in different directions. The ultrastructure of epithelial cilia and villa mostly sheds, causing damage and changes. The HNEPc were damaged and shed, as well as inflammatory cell infiltration was evident. After treatment, it was evident that the recovery of nasal cilia and nasal mucosal cell tissues in mice treated with Apt-BUD/C@MnO_2_ NFs was better. Eosinophils are closely associated the inflammatory state in AR. The infiltration of eosinophils causes a downstream series of inflammatory reactions, chemokine secretion, and inflammatory cell infiltration, thereby causing damage to the mucosa. Fig. 7H shows 100× and 400× magnification of the HE staining image of the nasal mucosa. The blue arrow in the enlarged image shows eosinophils. Compared to the control group, the HDM stimulation group showed significant infiltration of eosinophils, which was higher than that in the control group. The Apt-BUD/C@MnO_2_ NFs group showed a significant decrease in the number of eosinophils compared to that in the positive group (Fig. S9A). As shown in Fig. 7I, compared with the control group, the positive group in trachea of mice showed significant squamous epithelial metaplasia, while the Apt-BUD/C@MnO_2_ NFs group had significantly polarized epithelial cell arrangement and no metaplasia was observed. Goblet cells, a single-cell gland located between mucosal columnar epithelial cells, were primarily responsible for synthesizing and secreting mucin, forming a protective mucosal barrier. However, when type 2 inflammation is exacerbated, cytokines such as:IL-4 and IL-13 induce goblet cell hyperplasia and mucus production and contribute to fibrosis and reticular base membrane thickening thereby leading to tissue remodeling [33,34]. As shown in Fig. 7J, a large number of goblet cells were observed in the nasal mucosal epithelial cells of AR mice, and goblet cell proliferation was also observed in the BUD group alone. However, compared to the control group, the Apt-BUD/C@MnO_2_ NFs group did not show an increase in the number of goblet cells. These results were consistent with the number of goblet cells in the random field of view of the nasal slice (Fig. S9B). Previous studies have demonstrated that budesonide [35] exert a weak effect on goblet cell hyperplasia and mucus secretion inhibition. In the present study, we found that budesonide-loaded nanomaterials(Apt-BUD/C@MnO2 NFs) markedly suppressed the proliferation of goblet cells, with a superior efficacy compared with the budesonide monotherapy group. Accordingly, we conclude that Apt-BUD/C@MnO2 NFs can more effectively inhibit tissue remodeling and reverse the progression of inflammation in allergic rhinitis. Mitobiogenesis is a balanced process, occurring alongside mitochondrial clearance mechanisms, which together ensure that the cell maintains an optimal number of mitochondria [36]. Peroxisome proliferator-activated receptor gamma coactivator-1alpha (PGC-1α) and mitochondrial transcription factor A (TFAM) are important proteins involved in mitochondrial biogenesis and play a regulatory role in mitochondrial function. According to the immunohistochemical results in Fig. 7K, L, the expression of mitochondrial biogenesis related proteins PGC-1α and TFAM in the nasal mucosal epithelium of AR mice was lower than that in the normal control group. After treatment, the increased expression of PGC-1α and TFAM indicated that our nanozyme drug system can restore mitochondrial biogenesis and achieve AR therapy. Key regulatory factor for mitochondrial biogenesis, PGC-1α: it serves as the “master switch” for mitochondrial biogenesis (able to activate the expression of nuclear genes and mtDNA) and can also influence mitochondrial fission efficiency by regulating the activity of division-related proteins. When cellular energy is insufficient, the activation of AMPK kinase promotes mitochondrial biogenesis (increasing the cell's energy production capacity). TFAM is encoded by nuclear genes and, after entering the mitochondria, binds to mitochondrial DNA to participate in the initiation of mtDNA replication and transcriptional regulation, thereby maintaining the stability of mitochondrial DNA. Therefore, increased expression of both ensures normal mitochondrial quantity and function, reducing oxidative stress damage while enhancing the cell's energy supply. The unregulated expression of TFAM and PGC-1α illustrated that Apt-BUD/C@MnO_2_ NFs suppress the oxidative stress in nasal epithelium mucosa which caused by allergen and maintain cellular homeostasis.

In addition, no obvious tissue abnormalities or lesions were found in the heart, liver, spleen, lung and kidney of each group of mice (Fig. S10), indicated that our Apt-BUD/C@MnO_2_ NFs do not cause any biologically toxic in mice.

### 3.7 *In vivo* apt-BUD/C@MnO_2_ NFs suppress inflammatory response in AR and influence the gut bacterial microbiota which inhibits the progression of AR

**Fig. 8:**
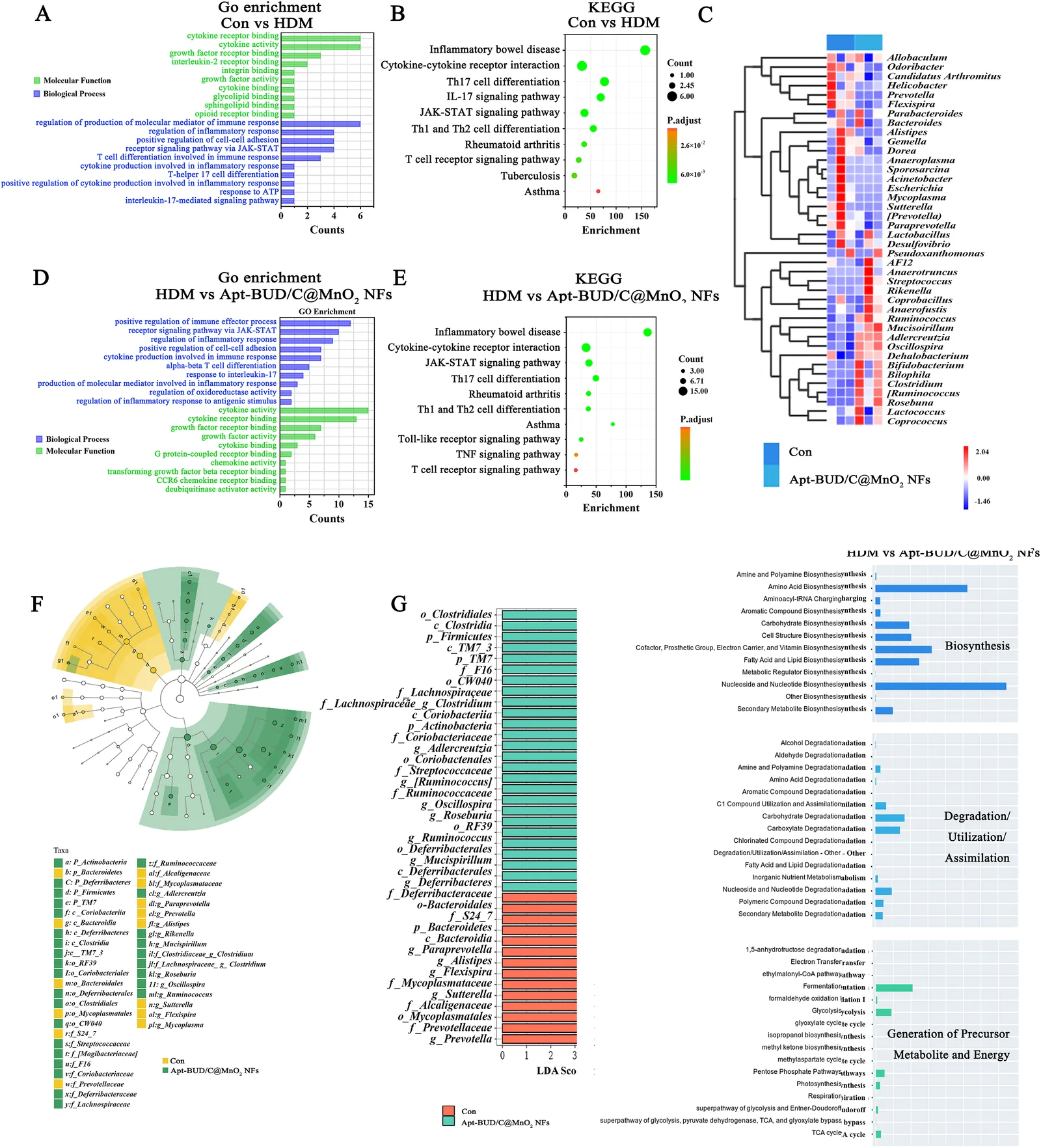
Enrichment of differential expressed genes in the cytokine antibody array. GO BP (A, D) and KEGG (B, E) enrichment analyses of differentially expressed cytokine genes detected in mouse serum of the three group (Control, HDM, Apt-BUD/C@MnO_2_ NFs). (C) Heatmap of microbial species in mouse feces of the two group (Control, Apt-BUD/C@MnO_2_ NFs). (F-G) LEfSe results on mouse feces microbiomes of the two group (Control, Apt-BUD/C@MnO_2_ NFs). (H)Metabolic pathway profiles of microbiota after Apt-BUD/C@MnO_2_ NFs treatment. Apt-C@MnO_2_ NFs is used in the figure legend to refer to the HDM + Apt-C@MnO_2_ NFs group.

Gene ontology analysis of differentially expressed genes identified in the antibody array in mouse serum for the control, HDM and HDM + Apt-BUD/C@MnO_2_ NFs groups was also performed. In the control and HDM groups, the Gene GO terms associated with the majority of DEGs, as shown in Fig. 8A, were predominantly related to the receptor signaling pathway mediated by JAK-STAT cell differentiation involved in the immune response, T-helper 17 cell differentiation and interleukin-17-mediated signaling pathway. The similar result was also observed in the HDM and Apt-BUD/C@MnO_2_ NFs groups. The GO terms associated with the majority of DEGs (Fig. 8D) were primarily involved in receptor signaling pathways via JAK-STAT, alpha-beta T cell differentiation, response to interleukin-17, CCR6 chemokine receptor binding, regulation of inflammatory response to antigenic stimulus, and regulation of oxidoreductase activity. KEGG pathway enrichment analysis of findings for DEGs in the antibody array of mouse serum between the control and HDM groups (Fig. 8B) revealed that they were primarily associated with the IL-17 signaling pathway, JAK-STAT signaling pathway, Th1 and Th2 cell differentiation, Asthma, Toll-like receptor signaling pathway. HDM and Apt-BUD/C@MnO_2_ NFs groups (Fig. 8E) revealed that the DEGs were mainly associated with Th17 cell differentiation, JAK-STAT signaling pathway, Th1 and Th2 cell differentiation, Toll-like receptor signaling pathway, and T cell receptor signaling pathway. In order to observe the differences in gut microbiota between the Apt-BUD/C@MnO_2_ NFs treatment group and the normal group of mice, fecal samples collected from mice of the two groups were submitted for bacterial microbiota profiling (Fig. 8C). The antibody array of mouse serum was carried out among the control, HDM and Apt-BUD/C@MnO_2_ NFs groups (Fig. S9A and B). The levels of pro-inflammatory cytokines (TNF-α) and Th2 related cytokines (IL-4) were increased in HDM group and decreased in the HDM + Apt-BUD/C@MnO_2_ NFs group(Fig. S11A and B), which is similar to the *in vitro* results in Fig. 5E–H.

LEfSe was applied to the microbiota data of Apt-BUD/C@MnO_2_ NFs and control mice feces (Fig. 8F–G), finding 40 differentially abundant taxonomic clades (a = 0.01) with an LDA score higher than 3.0. Our study found that in the two groups, the abundance of Bacteroidetes was generally consistent. In the Apt-BUD/C@MnO_2_ NFs group, two representative genera of Bacteroidetes: Prevotella and Bacteroides, had higher abundance (Fig. 8G). In AR patients, the abundance of Bacteroidetes decreased [37].It is believed that these bacteria may inhibit Th2-type inflammatory responses by regulating immune tolerance. Some short-chain fatty acid (SCFA) producing bacteria‌, such as species from the genera Roseburia and Bifidobacterium, showed significantly higher abundance in the Apt-BUD/C@MnO_2_ NFs group compared to the control group. Short-chain fatty acids have immunomodulatory functions, and the increased abundance of these bacteria can lead to more short-chain fatty acid production, thereby enhancing immune regulation [38]. The metabolic pathway shows the process of high-level amino acid biosynthesis and fatty acid and lipid biosynthesis. The amino acid biosynthesis, such as tryptophan catabolites (Fig. 8H).

The above results illustrated that, HDM induce Th2 and Th17 related complex inflammation and cause oxidative damage in HNEPc. However, the Apt-BUD/C@MnO_2_ NFs suppressed the inflammatory reaction and regulated ATP metabolism. Apt-BUD/C@MnO_2_ NFs increased abundance of these gut bacteria can lead to more short-chain fatty acid production, thereby enhancing immune regulation and occurrence of AR.

## 4 Conclusion

In this study, we developed a simple and replicable strategy for constructing a multifunctional nanozyme-drug synergistic therapy nanosystem, BUD loaded Apt modified carbon@manganese dioxide nanoflower (Apt-BUD/C@MnO_2_ NFs), which can target epithelial cells and is used for dual-mode imaging guided nanozyme-drug synergistic AR treatment. The material was composed of porous C nanospheres and MnO_2_ nanozymes with a synergistic ROS scavenging effect. In addition, it can effectively load BUD and achieve a sustained drug release. In particular, the modification of the Apt allows Apt-/C@MnO_2_ NFs to target nasal mucosal epithelial cells. *In vitro* experiments revealed that Apt-C@MnO_2_ NFs have the characteristics of targeting epithelial cells, drug delivery, multi enzyme activity and regulation of inflammatory factors and have an inhibitory effect on inflammation. *In vivo* experiments have shown that Apt-BUD/C@MnO_2_ NFs can restore the expression of mitochondrial related proteins.

Reduce the infiltration of inflammatory cells, regulate inflammatory pathways, restore the function of nasal cilia to resist external stimuli, alleviate AR symptoms, and have an imaging tracking function. Apt-BUD/C@MnO_2_ NFs treatment reshaped gut microbiota, enriching anti-inflammatory and SCFA-producing bacteria, and upregulating amino acid and fatty acid biosynthesis pathways. In summary, our multifunctional system showed significant anti-inflammatory effects and provides new ideas for future AR therapy.

## CRediT authorship contribution statement

**Leichao Zhang:** Investigation. **Longying Cong:** Writing – original draft, Software, Investigation, Data curation. **Yiyao Gao:** Investigation. **Wei Zhang:** Investigation. **Yiting Liu:** Investigation. **Lingyu Zhang:** Writing – review & editing, Supervision, Software, Methodology. **Qian Xiu:** Writing – review & editing, Supervision, Methodology, Funding acquisition.

## Declaration of competing interest

The authors declare that they have no known competing financial interests or personal relationships that could have appeared to influence the work reported in this paper.

## Data Availability

Data will be made available on request.
