# Supplementary material for: EpCAM aptamer modified carbon@manganese dioxide nanoflower for synergistic drug-nanozyme allergic rhinitis treatment
Source: Mater Today Bio. 2026 Aug 26;40:103588. doi: 10.1016/j.mtbio.2026.103588 (PMC13553565; doi:10.1016/j.mtbio.2026.103588)
Supplement: Supplementary file 1 [file mmc1.docx]

Epcam aptamer Modified Carbon@Manganese Dioxide Nanoflower for Synergistic Drug-Nanozyme Allergic Rhinitis Treatment

Leichao Zhang, Longying Cong, Yiyao Gao,Wei Zhang, Yiting Liu, Lingyu Zhang, Qian Xiu*

Prof Q. Xiu, Department of Otolaryngology-Head and Neck Surgery, China-Japan Union Hospital, Jilin University, 126 Xian Tai Street, Changchun, Jilin,130000,P.R. China. E-mail: xiuqian@jlu.edu.cn

Dr L.C. Zhang, Department of Pathology, China-Japan Union Hospital of Jilin University, 126 Xian Tai Street, Changchun, Jilin,130000,P.R. China

Dr Y.Y. Gao, Department of Scientific Research Center, China-Japan Union Hospital, Jilin University, 126 Xian Tai Street, Changchun, Jilin,130000,P.R. China

W. Zhang, Department of Chemistry, Northeast Normal University, 5268 Ren Min Street, Changchun, Jilin, 130024,P. R. China.

L.Y. Cong, Prof L.Y. Zhang, Department of Chemistry, Northeast Normal University, 5268 Ren Min Street, Changchun, Jilin, 130024,P. R. China.

**Appendix A. Supplementary material**

**Chemical and characterazation.**

Synthesis of PCNSs

1.8 mL polyacrylic (PAA) aqueous solution (0.2 g/mL) was added into 300 mL deionized water, stirred for 15 min, then added 72 mg zinc oxide (ZnO), shook and continued ultrasonic for 10 min until the solution was clarified. 600 mL isopropyl alcohol (IPA) was added to the mixture with a drop funnel, and was magnetically stirred overnight to form PAA-Zn NPs[1]. PAA-Zn NPs was centrifugally dried and calcined in a tube furnace at 900 ^o^C for 3 h to obtain PCNSs.

Synthesis of C@MnO_2_ NFs

In an oil bath of 80 ^o^C, 20 mg PCNSs dispersed in 5 mL aqueous solution was added drop by drop into 65 mL KMnO_4_ solution (0.01mol/L), stirred for 1h, and C@MnO_2_NFs were obtained after centrifugation and washing.

Synthesis of Apt-C@MnO_2_NFs

1 mg C@MnO_2_ NFs and 1 mg NH_2_-PEG-COOH were added into 1mL aqueous solution, mixed evenly by ultrasound, and mixed on a vertical mixer for 12 h. Then 1 mg EDC and 1mg NHS was added into the ice bath for 30 min. Then, Epcam aptamer containing amino groups (5 '- NH_2_CACTACAGAGGTTGCGTCTGTCCCACGTTGTCATGGGGGGTTGGCCTG - 3')[2] [2]was added into above solution, and placed in a vertical mixing apparatus on 4 ^o^C mix for the night. Finally, we centrifuged to obtain Apt-C@MnO_2_ NFs.

Gel electrophoresis

10 μL of Marker, C@MnO_2_ NFs, Epcam aptamer and Apt-C@MnO_2_ NFs were added to different wells, and then 2 μL 6×electrophoresis sample loading buffer was added to each test hole. The parameters of the electrophoresis instrument are 100 V, 120 mA at constant pressure. After 30 min, the position and strength of the molecules on the gel were recorded by the developer.

Scavenging of ·OH

FeSO_4_·7H_2_O and H_2_O_2_ were added to deionized water, then salicylic acid (SA) was mixed into the above mixed solution and stirred at room temperature for 30 min. Then different concentration of C@MnO_2_ NFs was mixed into the solution and stirred for 1h. In addition, control groups should be prepared. SA was dissolved in deionized water, and 3 groups of SA solution are taken respectively, and 1 μL H_2_O_2_ was added to one group and FeSO_4_·7H_2_O was added to the other group. The absorbance of the supernatant at 510 nm was measured by ultraviolet spectrophotometer. Finally, the scavenging ability of Apt-C@MnO_2_ NFs on ·OH was evaluated by comparing the absorbance of the supernatant.

·OH was firstly produced by Fenton reaction, and ·OH was captured by DMPO: 10 μL of H_2_O_2_ (3 %), FeSO_4_·7H_2_O (0.4 mmol/L), DMPO (0.1 mol/L) and sample or distilled water were quickly mixed. The electron paramagnetic resonance (ESR) spectrum of ·OH at 1 min was detected in quartz capillary.

Scavenging of O2·^-^

Riboflavin (20 μM), methionine (13 mM), and NBT (75 μM) were mixed in deionized water (20 mL) to produce a reactant with characteristic absorption at 560nm. Subsequently, different concentrations of C@MnO_2_ NFs were added to the reaction solution, and the photoreduction inhibition rate was determined after 15 min of ultraviolet irradiation. Test the absorbance of the supernatant of different samples. The ability of C@MnO_2_ NFs to remove O_2_·- was evaluated by comparing the absorbance value change.

Scavenging of H_2_O_2_

The absorbance values of the characteristic absorption peaks of H_2_O_2_ in the test samples of different concentrations were determined, and the produced O_2_ was detected at the same time.

Mucus barrier model was established

(1) Configuration of artificial mucus: 500 mg DNA, 250 μL 50% yolk emulsion, 250 mg mucin, 0.295 mg DTPA, 250 mg NaCl, 110 mg KCl, 1mL RPMI were added into 50 mL water, ultrasonic until completely dissolved, and preserved at 4 ^o^C.

(2) 5 g gelatin was added into 50 mL water and microwave until melted.

(3)800 μL liquid gelatin was added into two clean glass bottles, cooled and solidified. Then 1.2 mL mucus was added, and 100 μL (0.5mg/mL) Apt-C@MnO_2_ NFs and C@MnO_2_ NFs were added by a dropper at top, respectively. Taking photos every 10 min to record the phenomenon. After 40 min, removed the supernatant, heated slightly to melt the gelatin layer, and measured the ultraviolet absorbance of the gelatin layer.

Cell culture

Human nasal epithelial cells (HNEpCs) were cultured at 37 °C, in a 5% CO_2_ environment with Dulbecco’s modified eagle medium (DMEM) supplemented with 10 % fetal bovine serum (FBS).

*In vitro* cytotoxicity

First, HNEpCs was grown on 96-well plates for 24 h. Then, serum-free DMEM containing Apt-C@MnO_2_ NFs (100 - 6.25 μg/mL) was added, respectively. After 24 h, 10 μL MTT was added to each well, continued incubation for 3 h, and then the medium was removed and 150 μL DMSO was added. Absorbance was measured at 560 nm, the survival rate of HNEpCs was calculated using equation 1:

$C\text{ell }Viability(\%)=\frac{Abs_{test\text{ }cells}}{Abs_{control\text{ }cells}}\times100\%$ (1)

Intracellular ROS and Mitochondrial detection

In a confocal dish, HNEpCs was added and incubated for 24 h. After the culture medium was removed, 200μg/mL HDM prepared with serum-free culture solutionwas added except blank control group and incubated for 24 h. After that, Apt-C@MnO_2_ NFs dissolved in serum-free DMEM (25μg/mL) was replaced and incubated for 12 h and the other groups were replaced with serum-free DMEM. Then DCFH-DA (0.01mM) in serum-free DMEM and Hoechst33342 (1μg/mL) was added to each dish in darkness, and incubated for 30 min in sequence. For mitochondrial detection, the dye changed to green fluorescent Mito-tracker, and for mitochondrial membrane potential monitoring, only JC-1 was added. Finally, confocal laser scanning microscope (CLSM) was performed.

Determination of SOD activity, MDA content and protein chip

In the 6-well plate, HNEpCs was treated the same as above process before dying.The cells in different groups were lysed and detected according to the instructions of the SOD kit and MDA kit. And the supernatant was collected for testing on a protein chip.

ATP test

HNEpCs was planted in 12-well plates for 24 h. 200μg/mL HDM was added except the control group for another 24 h, then 5μg/mL BUD solution, 25μg/mL C@MnO_2_ NFs, 25μg/mL BUD/C@MnO_2_ NFs, and 25μg/mL Apt-BUD/C@MnO_2_ NFs were added for 12 h, respectively. The level of ATP was detected by he ATP kit.

Cell targeting experiment

HNEpCs and Hela cells were seeded in confocal dishes respectively, and incubated for 24 h. Then 25μg/mL of RITC/C@MnO_2_ NFs was added to HNEpCs; RITC/FITC/Apt-C@MnO_2_ NFs was added to HNEpCs and Hela cells, respectively. Then the cell nuclei were stained with Hoechet 33342 and imaged by CLSM.

Hemolytic Test

Centrifuge the fresh blood, suck out the serum and throw it away. Then the blood is dispersed with NaCl solution, and then centrifuged, repeated operation several times to make the fresh blood supernatant colorless and transparent. Then absorb the solution 200μL and dilute it with NaCl to 10mL for use. Finally, PCNSs was added to the nacL-washed blood to dilute the final PCNSs concentration from 400μg/mL to 6.25μg/mL. In addition, the washed blood was dissolved in 1 mL of water as a positive control and dissolved in 1 mL of NaCl as a negative control. The above nine groups of solution were incubated in a water bath at 37 ^o^C for 2 h. Finally, the supernatant was centrifuged for 10 min at a rotating speed of 6000 r/min, the status was recorded by taking photos, and the supernatant was placed in a quartz dish. The absorbance at the wavelength of 540 nm was tested by ultraviolet spectrophotometer, and the hemolysis rate was calculated by the formula according to the measured data.

*In vivo* experiment

All animal procedures were performed in accordance with the guidelines for Care and Use of Laboratory Animals of Northeast Normal University (202302034). C57BL/6J male mice aged 6-8 weeks with a mass of 18-20 g were selected and kept in an SPF environment for one week, and then randomly divided into 6 groups with 6 animals per group for different treatments: (1) Control, (2) HDM, (3) BUD, (4) C@MnO_2_ NFs, (5) BUD/C@MnO_2_ NFs, (6) Apt-BUD/C@MnO_2_ NFs.

The AR mouse model sensitized by dust mites (HDM) was constructed. In addition to the blank control group with 10 μL pure PBS solution, mice were intranasal with 10 μL PBS solution containing 1 μg HDM protein for 3 days, and then the mice were fed normally for 1 week after the sensitization process. Afterwards, mice were intranasal exposed to 10 μL of PBS in Control group and HDM (10 mg/mL) in other groups, respectively. Subsequently, the therapeutic groups were intranasal instilled with BUD (2 μg/mL), C@MnO_2_ NFs (10 mg/mL), BUD/C@MnO_2_ NFs (10 mg/mL), and Apt-BUD/C@MnO_2_ NFs (10 mg/mL), respectively. The treatment was repeated every day for 5 days. At day 15, blood was collected for protein chip and blood examination, and nasal mucous membrane for SEM, hematoxylin-eosin (H&E) staining, Periodic Acid-Schiff (PAS) stain and Immuohistochemical staining。

Histological analysis and immunohistochemistry

Paraffin sections were stained with HE and then observed by two independent physicians. All monoclonal antibodies (anti-PGC1α, anti-mtTFA, ) were purchased from Novus（CO，USA). Primary antibodies were applied for 24 hours at 4 ^o^C, and slides were rinsed 3 times with phosphate-buffered saline (PBS) prior to incubation with the appropriate secondary antibodies for 2 hours. After further washing, the slides were stained with diaminobenzidine (DAB; Maixin, Fujian, Fuzhou, China) and counterstained with hematoxylin. Image Pro-Plus 6.0 software was used to analyze images.

Figure S1. High resolution XPS spectra of C, O and Mn for C@MnO_2_ NFs.

Figure S2. UV-Vis spectra of (A) C@MnO_2_ NFs and (B) Apt-C@MnO_2_ NFs.

Figure S3. Zeta potential of NFs before and after modification.

Figure S4. TEM images of Apt-C@MnO_2_ NFs

Figure S5. Cell viability of Apt-BUD/C@MnO_2_ NFs with different concentrations on HNEpCs cells.

Figure S6. Green Mito-tracker stained mitochondria after different treatments.

Figure S7. Effects of different groups on ATP levels in HNEpCs. Statistical significance: *p < 0.05, **p < 0.01(compared with HDM group).

Figure S8. Different concentrations of Apt-BUD/C@MnO_2_ NFs on the hemolysis rate of red blood cell, the embedded Figure is the corresponding photo.

Figure S9. (A) The number of eosinophils in the random field of view in the H&E staining of nasal mucosa(left). (B)The number of goblet cells in random field in PAS staining of nasal mucosa(right).

Figure S10 H&E staining images of main organs of mice in each group.

Figure S11. Effects of HDM and Apt-BUD/C@MnO_2_ NFs on cytokine secretion in mouse serum of the three group (Control, HDM, Apt-BUD/C@MnO_2_ NFs).The relative expression levels of (A)IL-4,(B)TNF-α, were assayed using antibody arrays .Data are expressed as mean ± SEM. Group differences were evaluated using ANOVA.

Figure S12 (A,C) Hematoxylin and eosin(H&E) staining of nasal mucosa of mice (magniﬁcation, ×400 Bar indicates 100 µm.) (B,D) SEM images of mice: cilium of nasal epithelium. A,B:HDM group, C,D:HDM+Apt-BUD/C@MnO_2_ NFs group (E) Heatmap of epithelial inflammation,injury and remodelling in different groups. (F) The bar chart of GO-BP annotation enrichment of differentially expressed genes in different groups (HDM, HDM+Apt-BUD/C@MnO_2_ NFs groups). (G) Protein-protein interaction (PPI) network of molecules regulating eosinophil recruitment, epithelial inflammation, barrier injury and tissue remodelling.Apt-BUD/C@MnO_2_ NFs is used in the figure legend to refer to the HDM+Apt-C@MnO_2_ NFs group.

Following HDM challenge, evident pathological injuries were observed in mouse nasal mucosa as demonstrated by both H&E staining and scanning electron microscopy (SEM). In the HDM group, (Fig. S10 A,B) disruption of epithelial continuity, disorganized arrangement of epithelial cells, massive shedding and tilting of surface cilia, as well as submucosal oedema accompanied by extensive inflammatory cell infiltration could be detected. After intervention with Apt-C@MnO_2_ NFs(Fig. S10 C,D), epithelial structural integrity was restored, ciliary loss and cilial tilting were markedly alleviated, and the degree of mucosal oedema and inflammatory infiltration was reduced. As reported, downregulated expression of multiple CLDN family members triggers epithelial barrier disruption, which participates in AR pathogenesis in both human patients and experimental animal models[3]. Meanwhile, epithelial damage releases IL-33, a key upstream trigger of Th2 inflammatory cascades in AR, and IL-33 knockout in pollen-induced AR mice alleviates eosinophil infiltration, IgE secretion, Th2 cytokine release and allergic sneezing symptoms[4, 5]. Our results revealed that HDM stimulation markedly suppressed the expression of tight junction genes (TJP2/3, OCLN, CLDN4) and upregulated the epithelial injury marker IL33 as well as matrix metalloproteinases MMP2/6/13 associated with epithelial remodeling (Fig. S9 E), leading to disruption of the epithelial barrier. Intervention with Apt-C@MnO_2_ NFs reversed these aberrant gene expressions: the expression of tight junction-related genes was restored, while injury alarmins and pro-remodeling MMPs were inhibited. These findings indicate that Apt-C@MnO_2_ NFs alleviate HDM-induced nasal epithelial barrier damage, restore epithelial barrier function, and suppress epithelial-derived inflammation and mucosal remodeling.

GO biological process (Fig. S9 G) enrichment analysis revealed that the differentially expressed genes were predominantly enriched in eosinophil migration, eosinophil chemotaxis, granulocyte recruitment, chemokine-mediated signaling pathway, and the regulation of barrier permeability, highlighting the central roles of eosinophilic inflammation and epithelial barrier dysfunction in HDM-induced AR. Consistently, the PPI network(Fig. S9 F) separated candidate targets into two interconnected functional clusters: an inflammatory module composed of chemokines (CX3CL1, CCL2, CCL5, CCL26), alarmin IL33, eosinophil activation marker EPX, and remodeling-related MMPs; and an epithelial barrier module consisting of tight junction proteins TJP1/2/3, OCLN and CLDN4. CX3CL1 displayed extensive interactions with IL33, multiple chemokines, EPX and MMP13 within the PPI network, indicating its important function in mediating eosinophil recruitment in nasal mucosa,which serves as an important initiator of HDM-induced eosinophilic inflammation. Hub nodes including CCL2, CCL5 and MMP2 physically linked inflammatory signaling to tight junction components.

###### Reference

[1] S. Li, L. Zhang, X. Liang, et al., Tailored synthesis of hollow MOF/polydopamine Janus nanoparticles for synergistic multi-drug chemo-photothermal therapy, Chemical Engineering Journal. 378 (2019) 122175. [https://doi.org/10.1016/j.cej.2019.122175](https://doi.org/https://doi.org/10.1016/j.cej.2019.122175).

[2] Y. Song, Z. Zhu, Y. An, et al., Selection of DNA aptamers against epithelial cell adhesion molecule for cancer cell imaging and circulating tumor cell capture, Anal Chem. 85 (2013) 4141-9. <https://doi.org/10.1021/ac400366b>.

[3] K. Takano, T. Kojima, M. Go, et al., HLA-DR- and CD11c-positive Dendritic Cells Penetrate beyond Well-developed Epithelial Tight Junctions in Human Nasal Mucosa of Allergic Rhinitis, 53 (2005) 611-619.https://doi.org/10.1007/BF01174182.

[4] M. Christine, O. Nathalie, G.J.P.o. Jean-Philippe, The IL-1-like cytokine IL-33 is constitutively expressed in the nucleus of endothelial cells and epithelial cells in vivo: a novel 'alarmin'?, 3.10(2018) e3331.https://:doi.org/10.1371/journal.pone.0003331.

[5] Y. Haenuki, K. Matsushita, S. Futatsugi-Yumikura, et al., A critical role of IL-33 in experimental allergic rhinitis, 130 (2012) 184-194.e11.https://doi.org/10.1016/j.jaci.2012.02.013.
